# Adolescent sleep and the foundations of prefrontal cortical development and dysfunction

**DOI:** 10.1016/j.pneurobio.2022.102338

**Published:** 2022-08-11

**Authors:** Paul G. Anastasiades, Luisa de Vivo, Michele Bellesi, Matt W. Jones

**Affiliations:** 1 University of Bristol, School of Physiology, Pharmacology & Neuroscience, University Walk, Bristol BS8 1TD, UK; 2 University of Bristol, Translational Health Sciences, Dorothy Hodgkin Building, Whitson Street, Bristol, BS1 3NY, UK; 3 University of Camerino, School of Pharmacy, via Madonna delle Carceri 9 62032, Camerino, Italy; 4 University of Camerino, School of Bioscience and Veterinary Medicine, Via Gentile III Da Varano 62032, Camerino, Italy

**Keywords:** neurodevelopment, mental health, sleep disruption, synapses, glia, adolescence

## Abstract

Modern life poses many threats to good-quality sleep, challenging brain health across the lifespan. Curtailed or fragmented sleep may be particularly damaging during adolescence, when sleep disruption by delayed chronotypes and societal pressures coincides with our brains preparing for adult life via intense refinement of neural connectivity. These vulnerabilities converge on the prefrontal cortex, one of the last brain regions to mature and a central hub of the limbic-cortical circuits underpinning decision-making, reward processing, social interactions and emotion. Even subtle disruption of prefrontal cortical development during adolescence may therefore have enduring impact. In this review, we integrate synaptic and circuit mechanisms, glial biology, sleep neurophysiology and epidemiology, to frame a hypothesis highlighting the implications of adolescent sleep disruption for the neural circuitry of the prefrontal cortex. Convergent evidence underscores the importance of acknowledging, quantifying and optimizing adolescent sleep’s contributions to normative brain development and to lifelong mental health.

## 1 Introduction

**Figure 1:**
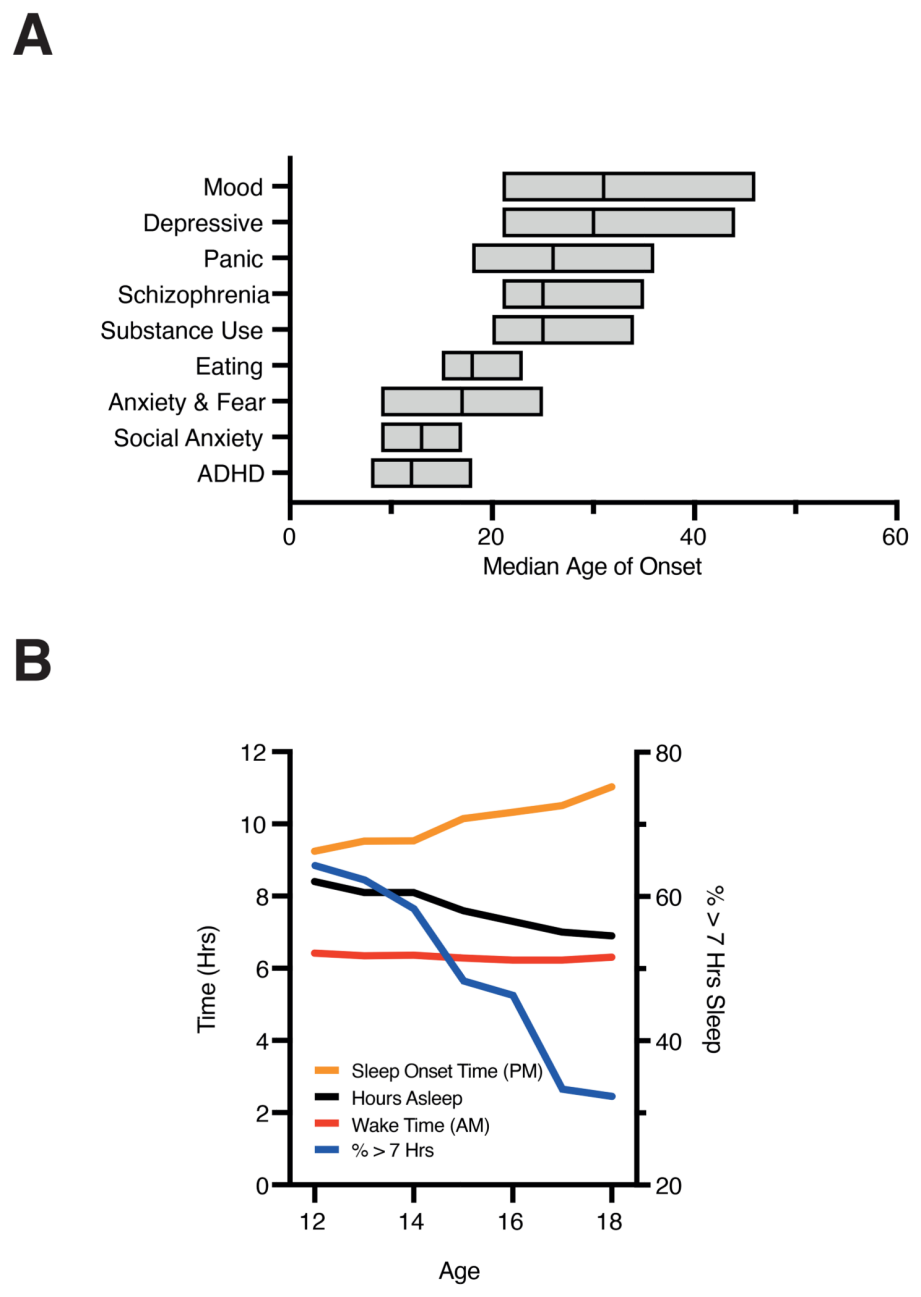
Changes in sleep patterns and neuropsychiatric disease onset during adolescence A) Age of onset for different mental diagnoses highlighting the emergence of clinical symptoms during, or shortly after the adolescent period. A spectrum of psychiatric, mood, and affective disorders begin in the teens and early twenties, just as the body and brain is in the process of transitioning from childhood to adulthood. Data shows median onset of various mental disorders with boxed region indicating 25th and 75th percentiles. B) A delay in evening sleep onset time across adolescence without a concomitant delay in morning wake time leads to an overall reduction in the average amount of sleep received by adolescents between the ages of 12-18. Consequently, there is a sharp reduction in the percentage of teenagers sleeping for at least 7 hours a night, despite typical teenagers requiring around 9 hours of sleep. Data in A replotted from Solmi et al., 2021 and in B from Keyes et al., 2015 and Carskadon, 2011.

Mental, neurological, and substance use disorders contribute 13% of the global burden of disease and are predicted to be the main cause of global mortality and morbidity by 2030 (World Health Organisation, 2011). The origin of many adult mental health problems includes atypical neurodevelopment driven by complex interactions between biological, environmental, and psycho-social factors (Paus et al., 2008). The adolescent transition to adulthood is particularly important, and coincides with presentation of several neuropsychiatric disorders (Blakemore, 2019; Solmi et al., 2021) (Fig. 1A). Adolescence is heralded by the onset of puberty and reflects a complex recipe of endocrine, somatic and nervous system developments (Appendix A), including sleep and circadian changes. Adolescents acquire an offset chronotype characterized by delayed sleep and rise times, leaving them misaligned with adult society and often misconstrued as “lazy”. This shifted chronotype means adolescent sleep becomes trapped between the biological drive for delayed sleep onset and societal demands for early waking (Tarokh et al., 2016). This issue has come to the fore recently following links between sleep loss and reduced academic performance (Beebe et al., 2017; Hysing et al., 2016; Urrila et al., 2017). However, as we discuss in this review, a more profound problem looms: compromised sleep may encumber normal adolescent brain development and increase the risk of lifelong mental health struggles.

Insufficient adolescent sleep is a growing global trend. Large (>250,000) cohort studies indicate that the proportion of adolescents regularly sleeping for >7h per night has decreased to less than 40% over the past 20 years (Keyes et al., 2015), while adolescents left undisturbed tend to sleep for >9h per night (Carskadon, 2011) (Fig. 1B). Epidemiological data consistently reveal that 18-year-old school pupils sleep 1-3h less per night than 12-year-olds, independent of country. This effect is likely exacerbated by features of modern society including caffeine, internet communication (Crone and Konijn, 2018), physical inactivity and socioeconomic disparities (Jarrin et al., 2014; Owens et al., 2014). Paralleling this reduction in sleep is a stark increase in the prevalence of adolescent mental health disorders, a trend not observed in older age groups (Twenge et al., 2019). Societal constraints on sleep-wake times may therefore add further pressure to adolescent mental well-being, already strained by concerns over global pandemics, climate change, racial discrimination, reduced job prospects and long-term insecurity. The consequences of such a burden are yet to unfold, but are clearly of immense societal significance given the huge reduction in quality of life and vast financial costs associated with the treatment and lifelong care of young people who develop mental health disorders.

It is therefore vital to integrate understanding of sleep’s contributions to both healthy and disordered adolescent brain development with mechanistic routes through which sleep disruption impacts adolescent mental health (Galván, 2020). To do so, we must identify brain hubs that are central to the aetiology of mental health disorders, impacted by sleep quality and quantity and particularly susceptible to insult during adolescent neurodevelopment. One lead candidate is the prefrontal cortex (PFC), which functions as the brain’s “central executive” and mediates a wide array of cognitive processes (Miller and Cohen, 2001). The PFC is associated with many of the afflictions that emerge during adolescence (Larsen and Luna, 2018) and functions as a key hub linking cortical and limbic brain regions implicated in cognitive and affective (dys)function (Goldstein and Walker, 2014; Krause et al., 2017). The PFC also receives dense neuromodulatory input from structures involved in regulating sleep and arousal, while suboptimal PFC function is a consistent symptom of the sleep-deprived brain (Krause et al., 2017). The adolescent PFC is also highly sensitive to psycho-social stress and associated signalling via the hypothalamic-pituitary-adrenocortical (HPA) axis (Appendix B). These factors all converge on an adolescent PFC undergoing intensive refinement of the connectivity scaffolded during childhood (Larsen and Luna, 2018; Sydnor et al., 2021). Emerging evidence suggests that disrupting prefrontal adolescent maturation may be uniquely damaging (Canetta et al., 2021), with profound implications for the numerous cortico-limbic networks routed through this central cognitive hub.

Triangulating across neurodevelopmental and cognitive neuroscientific and psychopathological domains prompts the hypothesis that the adolescent PFC, rendered vulnerable by immaturity and a flurry of interdependent neurodevelopmental changes, is highly sensitive to the cumulative consequences of chronic sleep disruption. Factors that limit adolescent sleep may therefore impede normal, healthy maturation of the PFC, and in some cases add to or amplify other sources of early-life adversity to cause enduring damage, placing teenagers at greater risk of lifelong cognitive and psychiatric impairment.

Can this hypothesis be definitively tested and mapped to causal mechanisms? Does the neurobiology of adolescence unveil aetiologies that bind sleep and sleep disruption to PFC development and psychopathology and, if so, what are the implications for lifelong health? In this review, we aim to provide mechanistic insights into how chronic adolescent sleep loss not only promotes acute lapses in cognition, affective status and psychotic episodes, but may also cause maladaptive, long-lasting network and cellular-level deficits. To do so, we first summarize epidemiological evidence linking adolescent chronic sleep disturbance with increased risks of developing neuropsychiatric disorders such as depression and schizophrenia. We then focus on the neurobiology of PFC development during adolescence and sleep’s central roles in its maturation, highlighting some of the effects of sleep disturbances at the cellular and network level. Finally, we propose opportunities for future research and treatment: measuring sleep and its hallmark brain activity opens a non-invasive window onto brain development, enabling early detection and personalized intervention at population-relevant scales.

## 2 Associations between adolescent sleep disruption and neuropsychiatry

When analysing sleep disturbances, interrelated metrics of both quantity and quality are key. Sleep quantity refers to the time spent asleep, while quality is harder to define and can be based on subjective report of how restorative sleep feels, and/or neurophysiological metrics such as slow-wave activity. Most studies of sleep disruption are in adults, and infer these metrics from questionnaires; standardization is limited, though the evolution of wearable devices is enabling more objective and longitudinal measures (Imtiaz, 2021) that may illuminate different strata of the umbrella diagnosis of “insomnia” (Crouse et al., 2021). While sleep ‘quality’ is more nuanced and challenging to quantify than ‘quantity’, it does appear to be the better measure of restorative sleep, and predictor of mental health status. Consensus, composite measures of “sleep health” incorporating both psychological and physiological measures would certainly advance the field, though measures may need tuning to capture specific aspects of sleep need and physiology at different ages; healthy sleep in a newborn would not be healthy in a pensioner.

Adolescence is characterised by shifting social relationships, increased emotional reactivity, risk taking and greater propensity for drug and alcohol abuse (Blakemore and Mills, 2014; Larsen and Luna, 2018; Spear, 2000). Even amongst otherwise healthy subjects, disrupted sleep seems to amplify the adolescent phenotype, with sleep deprived adolescents displaying elevated risk-taking behaviour including sexual risk-taking, alcohol and drug use (Lee et al., 2012b; Logan et al., 2018; Short and Weber, 2018; Weaver et al., 2018). A study of healthy college students who all obtained 7 hours sleep per night showed that sleep quality has a marked effect on measures of daytime sleepiness, physical and mental wellbeing (Pilcher et al., 1997). Poor sleep hygiene and consequential reductions in sleep quality also correlate with depression in students (Peach et al., 2016), while adolescents undergoing insomnia evaluations show strong links between various metrics of sleep quality and ADHD, conduct disorder, anxiety and affective problems (Van et al., 2019).

Depression and anxiety reflect the commonest mental health complaints in adolescents. Chronic periods of reduced sleep (Appendix B) negatively influence mood and emotional regulation in adolescents (Fuligni and Hardway, 2006; Lemola et al., 2013), while increased sleep has the opposite effect, reducing rates of anxiety and depression (Ojio et al., 2016) and predicting a lower likelihood of recent cigarette and marijuana use at 2-year follow-up (Pasch et al., 2012). Even a few nights of sleep restriction can impact adolescent cognitive performance and disposition, with effects increasing cumulatively despite weekend sleep catch-ups (Lo and Chee, 2020; Talbot et al., 2010). Sleep’s impact on the adolescent brain has been linked to the limbic system, which plays a key role in regulating affective status (Goldstein and Walker, 2014). Interestingly, adult insomniacs are more likely to re-engage limbic circuits during recall of emotional memories, a phenotype reminiscent of the limbic-dominated adolescent (Wassing et al., 2019).

Links between sleep and depression have been explored in longitudinal studies targeting pre- and peri-adolescent cohorts across the globe, revealing how self-reported sleep disturbances or reduced number of average hours spent sleeping per night during early adolescence predict instances of depression later in life (Alvaro et al., 2014; Gobin et al., 2015; Goldstone et al., 2020; Ojio et al., 2016; Orchard et al., 2020); cf Cheng et al., (Cheng et al., 2020). In the extreme, sleep loss and concomitant effects on mood increase suicide risk (Lee et al., 2012b; Liu, 2004; Weaver et al., 2018), with adolescents self-reporting less than 6 hours sleep per night three times more likely to consider suicide than those achieving 8 hours sleep (Weaver et al., 2018).

Sleep disruption also presents during premorbid stages of schizophrenia and strongly associates with psychotic experiences across healthy, at-risk and patient populations (Andorko et al., 2017; Davies et al., 2017; Hennig and Lincoln, 2018; Kahn-Greene et al., 2007; Lee et al., 2012a; Mayeli et al., 2021; Mulligan et al., 2016; S. Reeve et al., 2019). In adolescents at ultra-high risk (UHR) of developing psychosis, several sleep metrics predict the longitudinal course of psychotic symptoms (Lunsford-Avery et al., 2017, 2013). Turbulent sleep caused by nightmares, even if occurring many years prior, can portend both psychotic experience (Thompson et al., 2015) and suicidal ideation (Liu, 2004; Russell et al., 2018), highlighting the predictive capacity of sleep quality in the context of adolescent mental health (Oshima et al., 2010).

While links between sleep disturbances and neuropsychiatric symptoms are well documented (Koyanagi and Stickley, 2015; Kraepelin, 1919; Lovato et al., 2017; O’Callaghan et al., 2021), they were initially considered as secondary features of the disorders or side effects of medication (Reeve et al., 2015; Rehman et al., 2017). More recently a complex, bidirectional association has emerged. For example, the quality of the previous night’s sleep can influence the instance of positive (Mulligan et al., 2016; Reeve et al., 2015) and negative symptoms (Lunsford-Avery et al., 2013) in both psychotic patients and those at risk of schizophrenia. Amongst schizophrenia patients, those presenting with co-morbid clinical sleep disorders displayed elevated paranoia, hallucinations, cognitive disorganization, depression, and anxiety (Sarah Reeve et al., 2019). Recurring sleep disturbance in adolescents with affective disorders also indicates those most likely to relapse, or least likely to respond to treatment (Clarke and Harvey, 2012; J. Emslie et al., 2001).

Causal links are supported by observations that treatment with Cognitive Behavioural Therapy for Insomnia (CBTi) both improves sleep quality and reduces paranoia, hallucinations, anxiety and depression in clinical and non-clinical samples (Cunningham and Shapiro, 2018; Freeman et al., 2015; Waite et al., 2018). This broad therapeutic effect is significant given that sleep’s association with psychosis is partially mediated by negative affect (Hennig and Lincoln, 2018; Mulligan et al., 2016; Reeve et al., 2018; S. Reeve et al., 2019).

In summary, convergent evidence indicates that adolescent sleep impairment increases detrimental patterns of behaviour (Logan et al., 2018; Short and Weber, 2018; Weaver et al., 2018), predicts the emergence of mental health disorders (Goldstone et al., 2020; Lunsford-Avery et al., 2013; Orchard et al., 2020) and correlates with symptom severity and duration (Fuligni and Hardway, 2006; Lemola et al., 2013; Sarah Reeve et al., 2019). These observations support the hypothesis that impaired sleep negatively impacts adolescent brain function and, in severe cases, promotes neuropsychiatric symptoms with potentially lifelong consequences.

However, the range of sleep disturbances associated with poor mental health is wide and often derived from subjective, non-standardized evaluation of sleep quality, duration, and rhythmicity. Studies of objective sleep measures encompassing the period before and after neuropsychiatric symptom manifestation are limited, have small sample sizes, rarely target adolescents, and can report inconsistent results. Defining how adolescent sleep impairment contributes to neuropsychiatric disease requires large cohort longitudinal studies, including polysomnographic monitoring in children and adolescents before and throughout the course of the pathology. Although more work is needed to establish direct, causal mechanisms, there is mounting evidence that sleep has powerful influence over the adolescent brain. To gain greater insight, we must delve deeper into the neurobiology of adolescence and sleep’s central roles in developmental processes, particularly those involving the PFC.

## 3 Development of PFC functional networks and their sensitivity to sleep disruption

Although prefrontal networks are certainly not the sole casualties of adolescent sleep loss, they consistently rank amongst the most severely impacted (Krause et al., 2017; Lunsford-Avery et al., 2020) and impactful, since our brain’s cognitive capacities depend upon the PFC’s extensive interconnectivity with limbic and cortical regions, including the amygdala, hippocampus, default-mode network (DMN) and frontoparietal network (FPN) (Krause et al., 2017; Miller and Cohen, 2001). A recent brain-wide study of adolescents compared fMRI scans after a normal night’s sleep or a single night of acute sleep restriction performed a week later. Following sleep restriction, abnormal activity and functional connectivity were observed across limbic regions that connect to the PFC as well as components of the DMN and FPN (Robinson et al., 2018). These changes may represent compensatory shifts required to maintain normal function (Beebe et al., 2009), deleterious effects that contribute to changes in behaviour, or most likely a combination of both. Regardless of the underlying cause, such changes may disrupt ongoing development of prefrontal networks during the adolescent period. This is consistent with recent work in the Adolescent Brain Cognitive Development (ABCD) cohort, showing that unhealthy sleep in adolescents has widespread consequences for the development of core brain networks (Brooks et al., 2022).

**Figure 2:**
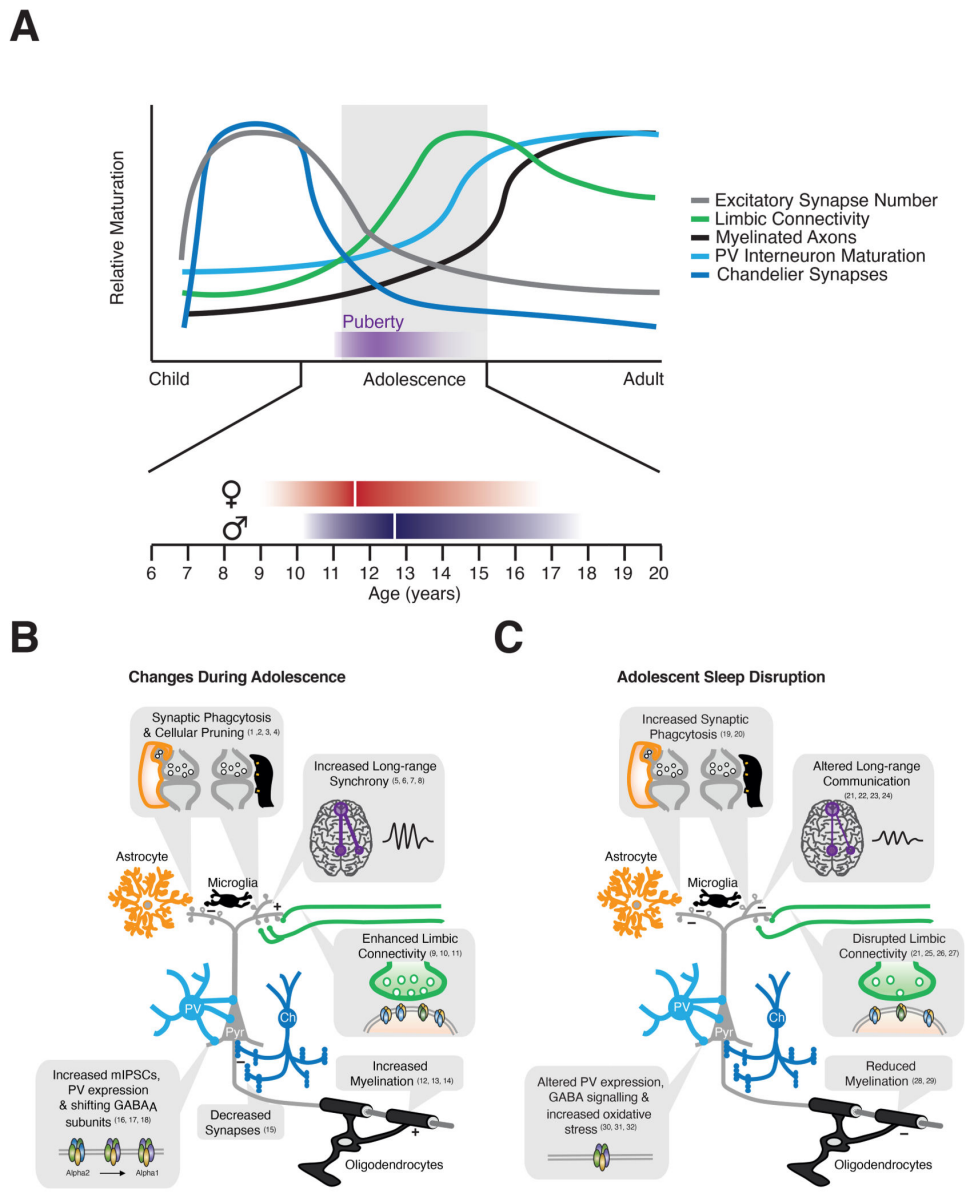
Adolescent network refinement and the impact of sleep A) Adolescence coincides with pronounced changes in multiple aspects of PFC development. Glutamatergic synapse density is strongly reduced, while limbic connectivity peaks during adolescence to produce a developmental phase where the limbic system has considerable influence over the PFC. Developmental changes in inhibition, followed by subsequent myelination lead to reduction in PFC plasticity and help stabilize PFC circuitry, including long-range coupling with components of the DMN and FPN, as we transition to adulthood. Sex differences manifest in PFC volume peak, thinning, and maturational coupling as well as in rates of neuronal apoptosis, and synaptic pruning. These sex differences are consistent with delayed PFC maturation in males relative to females which could in part reflect a parallel temporal shift in puberty onset (between the ages of 8 and 12 years in girls and between 9 and 14 years in boys; ~35 postnatal days in female rats, ~45 in males). B) Adolescent PFC maturation is characterized by refinement of intra-PFC and long-range connectivity. These changes bring about increased limbic connectivity, increased co-ordinated activity with other distal brain regions, such as components of the DMN and FPN. Changes in long-range interactions are thought to be driven, at least in part, by increases in axon myelination. At the synapse level changes in synaptic density, receptor subunit composition and function are observed at both excitatory and inhibitory synapses. Fast synaptic inhibition of PFC pyramidal neurons is promoted by the shift from alpha2- to alpha1-containing GABAA receptors, while chandelier synapses to the axon initial segment are reduced. Glutamatergic synaptic reorganisation is regulated by synaptic phagocytosis and associated pruning via astrocytes and microglia. C) Sleep impacts many of the developmental processes that occur during adolescence. Sleep disruption impacts glial cells, causing reduction in myelin thickness and increasing synaptic phagocytosis. Reduced sleep also causes oxidative stress in PV interneurons, altering PV expression levels and cellular function. Limbic and long-range connectivity are also impaired, uncoupling the PFC and other frontal cortices from connected brain regions, or forcing networks to work harder to maintain normal function. References: 1) Petanjek et al., 2011; 2) Mallya et al., 2019; 3) Drzewiecki et al., 2016; 4) Willing and Juraska, 2015; 5) Fair et al., 2009; 6) Sherman et al., 2014; 7) Tarokh et al., 2010; 8) Kurth et al., 2010; 9) Pattwell et al., 2016; 10) Benes, 1989; 11) Swartz et al., 2014; 12) Nagy et al., 2004; 13) Liston et al., 2006; 14) Sousa et al., 2018; 15) Anderson et al., 1995; 16) Gonzalez-Burgos et al., 2015; 17) Caballero and Tseng, 2016; 18) Hashimoto et al., 2009; 19) Bellesi et al., 2017; 20) Bellesi et al., 2015; 21) Robinson et al., 2018; 22) Beebe et al., 2009; 23) De Havas et al., 2012; 24) Tashjian et al., 2018; 25) Billeh et al., 2016; 26) Killgore, 2013; 27) Liu et al., 2016; 28) Telzer et al., 2015; 29) Bellesi et al., 2018; 30) Bridi et al., 2019; 31) Harkness et al., 2019; 32) Jones et al., 2019.

The DMN, which includes parts of prefrontal, parietal and cingulate cortex, supports introspective or stimulus-independent thought and correlates with cognitive task performance (Mason et al., 2007). Across adolescence, the DMN and FPN transition to support greater long-range integration at the expense of local connections (Fair et al., 2009). Consequently, resting DMN activity increases, mirroring FPN coupling (Fair et al., 2009; Sherman et al., 2014) and cognitive performance (Spear, 2000). Changes in functional connectivity may be linked to maturation of the underlying white matter, with working memory performance positively correlated with DMN myelination (Nagy et al., 2004). Similar shifts towards long-range integration are observed in rodent cortical networks (Nabel et al., 2020) while pathways linking the rodent PFC, amygdala and hippocampus are transiently upregulated during adolescence (Pattwell et al., 2016), mirroring elevated axon density and myelination seen in humans (Benes, 1989; Swartz et al., 2014) (Fig. 2A,B). Adolescent changes in cortical and limbic circuitry are supported at the level of functional connectivity (Arruda-Carvalho et al., 2017; Gee et al., 2013; Pattwell et al., 2016) and behaviour (Bos et al., 2015; Liston et al., 2006; Luna et al., 2001), further bolstering evidence that adolescence represents a formative period for the emergence of higher-order cognitive networks.

Sleep and wake states are hallmarked by distinct signatures of network activity that can also be used to measure the functional organisation of cortical networks. Sleep EEG reflects both genetic and environmental factors (Markovic et al., 2020) and, consistent with MRI studies described above, changes dramatically during adolescence (Tarokh et al., 2010). During non-REM sleep, slow-waves propagate through cortical pathways that overlap considerably with the DMN (Murphy et al., 2009). Slow-wave activity (SWA; EEG activity of 1-4.5 Hz) declines over the course of adolescence and its topography becomes weighted towards frontal regions as the cortex matures (Kurth et al., 2010). This correlates with strengthening of the DMN during healthy adolescence, suggesting that links between SWA and DMN status bear further investigation.

Sleep is an important regulator of the DMN, with DMN activity degraded following both chronic and acute sleep restriction (De Havas et al., 2012; Krause et al., 2017; Sämann et al., 2010). This is not simply due to sleep loss, as studies investigating habitual sleep patterns in adolescents and young adults suggest that sleep quality and circadian phenotype, rather than merely sleep duration, are the primary regulators of the DMN in this population. Poor sleep quality, but not total sleep time, in otherwise healthy adolescents correlates with reduced resting-state DMN connectivity (Tashjian et al., 2018), while regular sleep patterns are associated with decreased DMN path length, indicative of greater network efficiency (Lunsford-Avery et al., 2020). Moreover, adolescents and young adults with a late circadian phenotype show lower functional connectivity within the DMN relative to peers obtaining similar hours of sleep but with an early circadian phenotype (Facer-Childs et al., 2019).

Importantly, patients with persistent insomnia show abnormal patterns of cortical thickness within the DMN, which correlates with reduced executive function (Suh et al., 2016), linking sleep loss to structural and functional abnormalities within the DMN. Related findings are observed in rodents, where serial two-photon tomography quantifying brain-wide projections from high-order motor cortex in early adolescence revealed reduced structural connectivity in sleep-restricted mice, particularly for fibres projecting to limbic regions (Billeh et al., 2016). The extent to which such structural plasticity has functional implications and impacts different brain regions remains to be elucidated. Its dependence on neural activity also remains unclear, however silencing cortical neurons in the cingulate cortex during adolescence influences the refinement of long-range connections (Nabel et al., 2020). Sleep’s influence over adolescent networks may therefore in part be linked to changes in neural activity, a key driver of synapse formation and refinement (Andreae and Burrone, 2014; Bitzenhofer et al., 2021; González-Rueda et al., 2018; Pacheco et al., 2021). This hypothesis also suggests that the distinct neuronal activity patterns and neuromodulatory milieu present in REM and NREM sleep could differentially modulate PFC maturation. In sensory and motor cortices REM sleep has been proposed to shape preferentially synapse formation during early stages of development, whereas the role of NREM sleep is still controversial (Aton et al., 2009; de Vivo et al., 2019). More studies are needed to determine how REM and NREM sleep might differentially sculpt late maturing brain regions such as the PFC.

Neuromodulatory systems and the HPA axis may also contribute to the adolescent brain’s vulnerability to sleep loss. For example, preventing REM sleep for even a few hours can increase noradrenergic tone, causing hyperactivity of the affective salience network innervated by the locus coeruleus (Bellesi et al., 2016; Goldstein and Walker, 2014). Chronic REM sleep restriction in adolescent rodents is associated with increased anxiety and higher noradrenaline levels in the amygdala and hippocampus (da Silva Rocha-Lopes et al., 2018). Reduced sleep duration also reduced PFC inhibitory control of amygdala activity (Killgore, 2013; Krause et al., 2017) and PFC excitatory drive to the striatum (Liu et al., 2016), functionally reversing the developmental rebalancing that occurs within cortico-limbic circuits during normal adolescence. Sleep’s influence over the HPA axis is also significant given the unique susceptibility of adolescent limbic networks to stress (McCormick and Mathews, 2007) (Appendix B).

Sleep disruption’s influence over prefrontal networks is not unique to adolescence. However, what is distinctive is the significant maturational remodelling that occurs at this time (Gonzalez-Burgos et al., 2015; Larsen and Luna, 2018; Petanjek et al., 2011). This raises the question of precisely how sleep loss influences the maturation of PFC circuits and the potential for long-term dysregulation of PFC connectivity and function by sleep. Cortical development occurs sequentially, with lower-order sensory cortices reaching maturity prior to higher-order areas (Gogtay et al., 2004; Takesian and Hensch, 2013), culminating in a system that can support emergent functions. Development across the cortical hierarchy follows a characteristic maturational timeline which culminates in “critical” or “sensitive” periods of heightened plasticity, during which experience has unusually strong effects on brain circuitries and behaviour (Knudsen, 2004). Such sensitive periods are associated with numerous changes in cellular and synaptic properties, many of which occur in the adolescent PFC (Larsen and Luna, 2018) (Fig. 2A,B), consistent with adolescence encompassing a sensitive period for the formation of high-order cognitive circuits (Larsen and Luna, 2018). This raises the possibility that processes, including sleep loss, which disrupt prefrontal circuits during adolescence may cause lasting changes to network organization and function.

## 4 Sleep and the cellular mechanisms of circuit remodelling during adolescence

During adolescence, the PFC ceases being a net producer of dendritic spines - the anatomical substrate of glutamatergic synapses - and undergoes a period of synaptic pruning (Bourgeois et al., 1994; Petanjek et al., 2011) (Fig. 2A). This reduction in synapse number (Drzewiecki et al., 2016) has been linked to microglial and astrocytic phagocytosis (Chung et al., 2013; Mallya et al., 2019) and is conserved across humans (Petanjek et al., 2011), non-human primates (Anderson et al., 1995; Bourgeois et al., 1994) and rodents (Drzewiecki et al., 2016), contributing to waning PFC thickness during adolescence (Willing and Juraska, 2015). Consistent with the late maturation of the PFC, cortical thinning occurs in caudal (sensory) regions before frontal regions (Gogtay et al., 2004; Sowell et al., 1999).Sex differences have been observed in PFC volume peak, thinning, and maturational coupling in human longitudinal studies across adolescence (Lenroot et al., 2007; Raznahan et al., 2011, 2010). These studies highlight delayed but faster rates of PFC thinning in males compared with females, suggesting a late maturation of frontal subregions involved in impulse control, planning, and decision making in males. Adolescent synaptic remodelling also coincides with functional changes in synaptic proteins (Skene et al., 2017), pre-synaptic release machinery (Counotte et al., 2010) and neurite structure (Koss et al., 2014) that also differ between sexes and suggest a role for pubertal hormones in sex-specific adolescent PFC refinement.

The precise influence of sleep on synapse number and strength remains controversial and likely depends on both brain region and age (Bellesi and de Vivo, 2020; de Vivo et al., 2017; Zhou et al., 2020). In somatosensory cortex, spine pruning is enhanced during sleep in adolescent, but not adult, mice and is sensitive to sleep disruption (Maret et al., 2011). Sleep may also play dual roles in synaptic adaptations, selectively promoting the pruning of some spines and yet permissive to the formation, or potentiation, of others (Diering et al., 2017; Li et al., 2017). Such remodelling is predicted to enhance signal-to-noise ratios within cortical circuits (González-Rueda et al., 2018), potentially augmenting cognition and altering brain wide connectivity. Sleep/wake cycles also substantially modify the phosphorylation of synaptic proteins, affecting synaptic function and downstream regulatory networks (Brüning et al., 2019). Acute sleep deprivation in turn abolishes circadian rhythmicity in phosphorylation. Adolescent sleep may therefore support remodelling of synapse number, strength and the synaptic proteome within the developing PFC. Given widespread hormonal modulation of wake/sleep states throughout life (as reviewed in (Dorsey et al., 2021)), sexually dimorphic, sleep-dependent synaptic plasticity warrants further attention in future studies.

Functional shifts in GABAergic inhibition accompany the maturation of prefrontal excitatory synapses (Gonzalez-Burgos et al., 2015; Hashimoto et al., 2009) (Fig. 2A,B) and are driven by peripubertal hormonal changes, genetic factors, neural activity and diverse signalling cascades (Larsen and Luna, 2018; Piekarski et al., 2017). Studies to date have largely focused on fast-spiking, soma-targeting, parvalbumin positive (PV) interneurons, based on their association with neuropsychiatric disorders such as schizophrenia and autism (Dienel and Lewis, 2018; Hashemi et al., 2017) and notable ability to regulate developmental plasticity (Takesian and Hensch, 2013). PV expression, a good proxy for interneuron maturity (Anastasiades et al., 2016), increases in the developing PFC across species (Caballero and Tseng, 2016) (Fig. 2A) paralleling adolescent remodelling of glutamatergic inputs onto PV cells, but not other interneurons (Wang and Gao, 2009). A subpopulation of PV cells, termed chandelier cells, also undergoes refinement of their characteristic axo-axonic terminals across adolescence, mirroring reductions in excitatory synapses (Anderson et al., 1995) (Fig. 2A). As in sensory regions, the development of PV cells is influenced by both the external environment and intrinsic factors, which include social interaction, dopamine receptor expression and pubertal hormones (Bicks et al., 2020; Clemens et al., 2019; Piekarski et al., 2017; Tomasella et al., 2018; Tseng and O’Donnell, 2007).

Inhibitory transmission is highly dynamic throughout the sleep wake cycle, causing associated shifts in cortical excitation/inhibition balance (Bridi et al., 2019) and implicating sleep in the recalibration of inhibitory circuits. However, the influence of sleep loss on cortical inhibitory networks has been far less studied than excitatory circuits, limiting our ability to draw definitive conclusions. Tentative evidence suggests sensitivity to the oxidative stress-inducing effect of sleep disruption, with rats subjected to 6 and 12 hours of sleep prevention displaying particularly elevated levels of oxidative stress markers in PFC PV interneurons (Harkness et al., 2019). Studies of juvenile prairie voles show that NREM sleep fragmentation and REM sleep deprivation during periods where PV levels are normally upregulated caused persistent shifts in PV expression and social behaviour (Jones et al., 2019). Taken in the context of PV cells role as regulators of cortical developmental plasticity, accumulated oxidative stress caused by chronic sleep loss could lead to long-term impairment of network formation.

Neuromodulators are universal regulators of neural activity and plasticity during sleep and arousal. Studies from sensory cortices indicate that neuromodulators play important roles in regulating developmental plasticity (Takesian and Hensch, 2013; Yaeger et al., 2019). Consequently, adolescent shifts in neuromodulatory innervation, or receptor expression, may impact PFC development. Such changes have been studied most extensively in the context of mesocortical dopamine, motivated by changes in adolescent impulsivity, risk-taking, drug-taking and the emergence of neuropsychiatric disorders linked to abnormal PFC dopamine levels, such as schizophrenia (Arnsten et al., 2017; Larsen and Luna, 2018; Walker et al., 2017). PFC dopamine signalling peaks during adolescence, driven by exuberant ventral tegmental area axons (Rosenberg and Lewis, 1995) and transient upregulation of dopamine receptor expression (Tseng and O’Donnell, 2007; Walker et al., 2017). Elevated dopamine signalling during adolescence may also influence sleep-dependent network re-balancing. VTA neurons are involved in the generation of PFC “up-states” during anaesthesia (Tseng and O’Donnell, 2005), while dopamine has been implicated in hippocampal SWRs (Miyawaki et al., 2014), which play a key role coordinating PFC (and hippocampal) ensemble activity during both sleep and wakefulness. In support of dopamine’s role in adolescent network refinement, deletion of the dopamine D2-receptor produces specific deficits in adolescent synaptic pruning (Y.-Q. Zhang et al., 2021). Given the observed upregulation of PFC dopamine signalling during adolescence, these findings support a role for dopamine in PFC network maturation; sleep’s contributions to this role should be further explored.

In summary, these findings indicate that changing glutamatergic synapse composition and number, GABAergic connectivity, and neuromodulation converge upon the adolescent PFC, where they underpin the refinement of PFC circuitry that occurs at this time. However, neural circuits are also contingent upon glial partners, with microglia, astrocytes and myelination all contributing to synaptic and circuit refinement – and all at least partially dependent on sleep physiology and status.

Synapses are the main substrate for network level changes in both healthy aging and disease. However, rapidly expanding evidence implicates nonneuronal cells in both network refinement (Chung et al., 2013; Mallya et al., 2019; Sipe et al., 2016), sleep homeostasis (Davla et al., 2020; Haydon, 2017) and disease pathology (Bernstein et al., 2015; Rajkowska et al., 2002). Microglia and astrocytes contribute to developmental pruning and reorganisation of excitatory synapses via phagocytosis and release of synaptogenic molecules (Paolicelli et al., 2011; Stevens, 2008). Moreover, myelination is one of the final steps in network development, functioning as a key “breaking factor” (Larsen and Luna, 2018). As oligodendrocytes envelop axons with myelin they not only increase conduction velocity, but also prevent further branching and plasticity. A recent meta-analysis found that, as observed for cortical thinning, myelination was delayed in PFC development relative to other cortices (Sousa et al., 2018). Although the precise timeline of myelination varied across individual axon tracts, or for different measures of myelination, it increased steadily throughout adolescence before stabilizing during the 3^rd^ decade of life (Fig. 2A). Magnetic resonance studies of sexual dimorphism in myelination are mixed, reporting, on one hand, faster white matter growth in boys than in girls (Perrin et al., 2008), and on the other hand no significant difference in g-ratio, the ratio between axonal and fibre calibre, between sexes (Berman et al., 2018; Cercignani et al., 2017). However, animal studies reported higher levels of myelin basic protein and myelin proteolipid protein in female rat ventral PFC relative to males, together with greater density of projections from the ventral PFC to the dorsal striatum (Bayless and Daniel, 2015). These sex differences support the hypothesis that females may develop more PFC projections to the dorsal striatum than males, which could contribute to sex differences in inhibitory control (Bayless et al., 2013), though the pre-pubertal actions of neonatal gonadal hormones may also shape these developmental dimorphisms (Darling et al., 2020).

In summary, these findings indicate that changing glutamatergic synapse composition and number, GABAergic connectivity, neuromodulation, glial synaptic remodelling, and myelination converge upon the adolescent PFC, where they underpin the refinement of PFC circuitry that occurs at this time. However, neural circuits are also contingent upon glial partners, with microglia, astrocytes and myelination all contributing to synaptic and circuit refinement – and all at least partially dependent on sleep physiology and status.

## 5 Potential mechanistic links between sleep-dependent PFC development and psychiatric disorders

Glutamatergic, GABAergic, glial and neuromodulatory mechanisms are consistently implicated in most forms of mental illness, hence their disruption may bridge adolescent sleep to psychiatric outcomes. Deficits in glutamatergic signalling have been proposed as a core feature of schizophrenia, with reduced PFC spine density consistently observed in autopsy tissue from patients (Glausier and Lewis, 2013). Reduced prefrontal synapses have also been linked to depression (Holmes et al., 2019; Kang et al., 2012). Numerous genetic risk factors for depression and schizophrenia are expressed in cortical excitatory neurons (Bhattacherjee et al., 2019; Skene et al., 2017), with the levels of many of these genes dynamically up or down regulated during adolescence (Bhattacherjee et al., 2019; Skene et al., 2017). Rodent studies also suggest that deficits in prefrontal glutamatergic signalling may underly depressive symptoms (Joffe et al., 2020). Given the extensive glutamatergic refinement at play in the adolescent PFC, sleep disruption’s effects on these mechanisms warrant systematic testing.

Interneuron dysfunction is also a feature of many PFC-associated disorders (Dienel and Lewis, 2018; Hashemi et al., 2017). Schizophrenic patients show reduced levels of glutamate decarboxylase (GAD – the enzyme that catalyses GABA production), PV expression, chandelier cell synapses and circulating cerebrospinal GABA levels (Dienel and Lewis, 2018). In keeping with these findings, gamma oscillations, which critically depend on the balanced function of PV interneurons, are abnormal in schizophrenia patients (Uhlhaas and Singer, 2010), potentially due to disrupted NMDA-R and dopamine D2 receptor signalling (Carlén et al., 2012; Tomasella et al., 2018). Patients with major depressive disorder and bipolar depression also show deficits in GAD, GABA_A_ and GABA_B_ receptors, particularly in cortico-limbic networks involving the PFC and hippocampus (Fogaça and Duman, 2019; Luscher et al., 2011). Similar findings are observed in rodent models of depression, which reveal marked reduction in excitatory inputs onto PV cells. Depressive symptoms could be directly phenocopied in otherwise healthy mice by selective chemogenetic inhibition of prefrontal PV cells, supporting a direct causal relationship (Perova et al., 2015). Sleep deprivation increases oxidative stress on PFC PV cells (Harkness et al., 2019), again providing evidence of mechanistic overlap between the effects of sleep disruption and the pathophysiology of psychiatry disorders.

The activity of both glial populations is dynamic across the sleep/wake cycle and sensitive to sleep disturbances: microglial activation and synaptic phagocytosis are elevated in the brains of chronically sleep deprived adolescent mice (Bellesi et al., 2017), while astrocytes promptly upregulate hundreds of transcripts to engage specific cellular programs in response to wakefulness (Bellesi et al., 2015). Astrocyte-synapse interactions are elevated during periods of extended wakefulness (Bellesi et al., 2015), with acute sleep loss causing astrocytic phagocytosis of axon terminals in the adolescent mouse frontal cortex (Bellesi et al., 2017). This persists after chronic sleep restriction and is further accompanied by microglial activation and phagocytosis of synaptic structures (Fig. 2B,C). Thus, glial cells may promote aberrant pruning of cortical synapses in response to insufficient sleep, potentially mediating homeostatic removal of synaptic elements rendered dysfunctional by increased wakefulness; once again, its late maturation may render the PFC particularly vulnerable. These findings are consistent with reduced grey matter volume in patients with insomnia (Suh et al., 2016), reduced dendritic spines in the PFC of patients with schizophrenia and depression (Glausier and Lewis, 2013; Holmes et al., 2019; Kang et al., 2012), and neuroinflammatory theories of mental health disorders (Najjar et al., 2013).

Hence, PFC microglial activation and aberrant synaptic engulfment triggered by chronic sleep loss during adolescence might directly link adolescent disrupted sleep to dysfunctional PFC and increased risk of developing schizophrenia and depression, as suggested for other developmental insults (Schalbetter et al., 2022; Wohleb et al., 2018). Future studies should test this hypothesis in animal models and consider the interaction between sex hormones and sleep disruption in modulating microglial dependent synaptic pruning and the associated enduring effects on cognition and mental health.

As observed for other glial cell types, oligodendrocytes respond rapidly to sleep deprivation, downregulating genes involved in myelination and lipid turnover (Bellesi et al., 2013; Cirelli et al., 2004). This may explain sleep’s detrimental influence over myelination status (Fig. 2B,C). In adults, a single night of acute sleep deprivation can lead to reduced measures of white matter microstructural integrity (Elvsåshagen et al., 2015), while in adolescents variable sleep patterns correlate with reduced myelination in follow-up-scans at 18 months, including many pathways linking prefrontal and cingulate cortices with the FPN, limbic and thalamic regions (Telzer et al., 2015). Similar effects are observed in rodents, where chronic sleep restriction reduced myelin thickness in adolescent mice (Bellesi et al., 2018).

Myelination levels across fronto-parietal connections are associated with resilience to some of the negative cognitive effects of sleep deprivation (Cui et al., 2015). Combined with sleep’s ability to downregulate white matter integrity (Elvsåshagen et al., 2015; Telzer et al., 2015), this further suggests that adolescent networks may be particularly sensitive to sleep loss. Not only will sleep deprivation have strong additive effects on myelination, but adolescents may be inherently less able to buffer the negative cognitive impact due to lower myelin levels in late-maturing prefrontal and limbic networks (Sousa et al., 2018). Sleep’s command over myelination is also clinically significant. Hypomyelination is observed in patients with major depressive disorder (Sacchet and Gotlib, 2017) and schizophrenia (Maas et al., 2017), especially in axon tracts linking the PFC with cortical regions (such as the FPN) and limbic structures (Sacchet and Gotlib, 2017). Prefrontal myelination deficits pre-date symptoms in those at UHR of schizophrenia (Vijayakumar et al., 2016) and are also observed in the brains of asymptotic relatives (Camchong et al., 2009). It is possible to envisage how sleep disturbances could accentuate such pre-existing deficits, hindering the termination of adolescent plasticity, delaying the transition to adult-like cognition and even accelerating the advance of clinical symptoms in UHR groups.

In summary, while definitive links between sleep and mechanisms of synapse development remain to be systematically interrogated, sleep is positioned to play important roles in influencing the normal refinement of glutamatergic and GABAergic signalling that occurs during adolescence (Fig. 2A-C). Such changes may disrupt prefrontal excitatory/inhibitory balance (Bridi et al., 2019), drive abnormal synapse development, oscillatory activity and consequently influence network function and associated cognitive processing, as observed in adolescent disorders that impact the prefrontal cortex.

## 6 Enduring impacts of adolescent sleep disruption?

Early-life adversity and trauma are established risk factors in neuropsychiatry, with long-lasting influence over mental health outcomes (Ehlert, 2013). Although less traumatic than many other life events experienced by adolescents, reduced sleep is inherently stressful (Appendix B), meaning sleep is one of several factors powerfully positioned to influence the development and function of the adolescent brain at this critical time. Sleep and/or circadian disturbances feature in almost all brain disorders, often predating symptoms in adolescents who go on to develop generalized anxiety disorder, depression or schizophrenia (Baglioni et al., 2011; Orchard et al., 2020). Furthermore, several sleep and circadian metrics are under genetic influence (Dauvilliers et al., 2005), consistent with an underlying genetic predisposition for many of the disorders that inflict upon the adolescent brain. This is consistent with emerging evidence for some shared genetic architecture between sleep phenotypes and psychiatric disorders (O’Connell et al., 2021).

Sleep disruption may also occur concomitantly with other risk factors, and effects of sleep may be further compounded by complex interrelationships between the two, with trauma and stress often reducing sleep and consequently increasing deleterious patterns of behaviour that can expose adolescents to additional stressors or situations likely to negatively impact their well-being. Although many of these observations emerge from human studies and are largely correlative, they are supported by a growing body of animal experiments probing sleep’s role in brain maturation (Bellesi et al., 2018; Li et al., 2017; Maret et al., 2011). By integrating these findings with theories of cortical development, such as sensitive periods of plasticity, a framework emerges for understanding sleep’s impacts on the adolescent brain. This framework is broadly consistent with sleep’s known ability to influence sensitive/critical periods in other brain regions (Frank, 2017). Such a model is directly testable in humans, with further mechanistic insight available from laboratory animal studies.

One major prediction of this framework is that adolescent sleep disruption can culminate in enduring impacts on the structure and function of the PFC and connected brain regions. Proving this will require both longitudinal cohort studies integrating structural and functional studies of the PFC with objective sleep measures, such as polysomnography or actigraphy (Jalbrzikowski et al., 2021) and animal studies enabling high-throughput, controlled interrogation of the cellular mechanisms that underlie any observed changes. To date there has been relatively little research on this topic, however some studies highlight the feasibility of this approach and provide causal evidence that adolescent insults may elicit long-term deficits to PFC structure and function.

In rodents, adolescent – but not adult – social isolation causes reduced PFC myelin, which fails to recover once mice are reintroduced into a normal social environment (Makinodan et al., 2012). This effect was phenocopied by genetic manipulation of neuregulin signalling in oligodendrocytes (Makinodan et al., 2012), exemplifying how genetic and environmental factors may interact, for example in UHR groups discussed earlier in this review. Primates exposed to juvenile social isolation also show working memory deficits that last into adulthood (Sánchez et al., 1998), while rodents exposed to adolescent stressors display increased anxiety and abnormal social behaviours as adults (Gomes and Grace, 2017). At the circuit and systems level, adolescent PFC lesions cause increased depression-like behaviours in rodents and reduce VTA dopamine neuron activity (Uliana et al., 2020). Selectively silencing adolescent refinement of top-down circuits linking cingulate and visual cortices causes deficits in adult visual attention (Nabel et al., 2020), while silencing prefrontal PV neurons produces lifelong impact on synapse refinement and behaviour (Canetta et al., 2021). In the latter case the authors predict that any manipulation influencing PV activity during adolescence, such as oxidative stress (Harkness et al., 2019), would produce long-term deficits to the PFC. This prediction is consistent with recent findings exploring adolescent reduction in PV expression in the PFC (Caballero et al., 2020). In many of these studies, manipulations were also applied to adults but failed to cause pronounced deficits, consistent with our hypothesis of heightened vulnerability within the adolescent PFC. The short developmental timeline of rodents, coupled with the ability to manipulate the rodent brain using techniques such as chemogenetics will allow direct testing of causal mechanisms behind the deleterious effects of adolescent sleep loss. These experiments can also be performed in genetic models of neurodevelopmental disorders, to test how different genetic backgrounds influence sleep-mediated effects on the adolescent PFC.

The findings discussed in this review highlight how the course of adolescence has lifelong impact. Although there is much that remains to be discovered, reduced – and particularly poor quality – sleep during adolescence may have enduring consequences, given sleep’s central roles in brain maturation and mechanistic links to many of the key developmental processes that characterize the neurobiology of adolescence. In those at risk for neuropsychiatric disorders, this may be sufficient to initiate a self-reinforcing cycle that spirals towards disease. For example, it is possible to envision how sleep loss could negatively influence mood and affect in at-risk adolescents, increasing anxiety, further reducing sleep and ultimately triggering psychotic episodes, through combined effects on sleep and affective status (Hennig and Lincoln, 2018; Mulligan et al., 2016b; Reeve et al., 2018; S. Reeve et al., 2019). Additive genetic, environmental and sleep-dependent mechanisms may therefore combine to cause significant and lifelong deficits in PFC connectivity and function. Although we have largely focused on the manifestation of clinical symptoms, these findings also suggest that even otherwise healthy adolescents enduring general reduction in sleep quality or quantity may still suffer from suboptimal maturation of the cognitive and social brain, with clear societal implications (Blakemore and Mills, 2014).

## 7 Conclusions and future directions

We have outlined how sleep mediates or modulates many neurodevelopmental processes required for the adolescent refinement of PFC connectivity and function. While few features of adolescent brain development are entirely dependent upon sleep, all benefit from sleep and most are jeopardized by its disruption. However, the significant maturational remodelling that occurs in the adolescent PFC and its connected regions confers heightened vulnerability to sleep disruption. The late development of PFC and its central roles in adaptive behaviour throughout subsequent life also mean even relatively subtle disruption of PFC maturation may have enduring negative consequences. We have documented possible mechanistic links between altered adolescent sleep and increased risk of developing depression and schizophrenia, highlighting the neuronal and glial processes (a) impacted by reduced sleep quantity and quality and (b) implicated in psychiatric etiology.

At present, adolescence remains understudied: more synaptic- and cellular-resolution studies of adolescent brains are required to map the mechanisms of normal PFC development and the effects of sleep disruption, though the challenges of accessing human tissue continue to necessitate back-translation into other species; the conserved biology of adolescence makes this viable. It will also be important to consider sex as a biological variable given numerous sex specific differences in adolescent development, sleep and the prevalence of adolescent mental health disorders (Shansky and Murphy, 2021).

One important consideration is the well-documented differences in the organisation of frontal cortices across species, with rodents lacking regions found in primates (Laubach et al., 2018; Preuss and Wise, 2021). The homologous regions shared between rodents and humans are nonetheless of clear clinical significance (Preuss and Wise, 2021) and rodents are powerful models for dissecting causal mechanisms of disease (Homberg et al., 2021). Animal studies can be tightly controlled and offer detailed insight into the role of sleep deprivation on the PFC. The shorter maturational timeline of mice and rats allows for rapid, high-throughput testing using cutting-edge techniques to monitor and manipulate the brain during cellular, systems and cognitive level analysis. Findings can be extended to non-human primates to further enhance translational validity. What questions should we therefore aim to answer with these tools?

Firstly, it is evident that sleep does not solely impact the adolescent brain, yet the data reviewed here make the prediction that the adolescent brain would be more susceptible to sleep deprivation than the adult, yielding greater long-term deficits. Secondly, we would predict that those regions undergoing heightened plasticity, including the PFC, would be preferentially impacted by adolescent sleep disruption. Therefore, sleep disruption during adolescence should promote greater defects in executive control, as opposed to sensory perception or motor function. Finally, given that many of the deficits observed after sleep loss show co-morbidity with neurodevelopmental disorders, we would predict that rodents harbouring known mutations associated with these disorders would show greater sensitivity to the deleterious impact of sleep loss. Such studies are of considerable importance, as sleep and/or circadian disturbances feature in almost all brain diseases.

Sleep offers a unique window into adolescent brain development, enabling “population neuroscience” studies in humans to complement mechanistic studies in animals. Questionnaires and actigraphy constitute scalable and longitudinal estimates of sleep behaviour, while scalp EEG affords non-invasive, objective metrics of the development of neural homeostasis and prefrontal circuit activity and function. The advent and evolution of wearable actigraphic, metabolic, EEG and “smart-home” monitoring devices stands poised to offer substantive advances in the field, and should be used to inform standardized, composite measures of “sleep health” that are more robust and sensitive than current quantity/quality modalities. Combining these metrics with genetics in sizeable cohorts allows definition of the genetic architecture of brain development, circuit function and disease.

Sleep’s predictive power also presents important opportunities for early detection and targeted intervention should sleep patterns or quality go awry. Pragmatic, affordable and low-risk approaches such as CBTi may mitigate against the damage done by chronic adolescent sleep disruption. For example, there is some evidence that parents setting bedtimes of 10:00pm can reduce the risk of depression during later adolescence (Gangwisch et al., 2010). ‘Generation Z’ are facing a battery of new challenges and uncertainties, which have been further exacerbated by COVID-19 (Appendix C). Sleep is a key contributor to their capacity for resilience, with emergent evidence indicating that PFC network integrity – which depends on sleep quality – can be protective, while sleep disruption increases vulnerability (Heissel et al., 2018; Miller et al., 2018).

Recent randomized control trials quantifying the effects of melatonin or CBTi-based sleep therapies on primary measures of sleep quality and secondary measures of well-being have demonstrated feasibility, safety and some promising beneficial effects in children and adolescents, including those suffering ADHD, autism spectrum disorder and psychotic experiences (Freeman et al., 2017; Schroder et al., 2019). New transdiagnostic approaches are also currently being trialled to treat adolescent sleep disorders (Harvey, 2016). It will be critical to establish whether early targeting of sleep quantity and/or quality produces enduring beneficial effects, and how these therapies impinge upon the PFC mechanisms central to cognitive maturation.

More focused, neurobiologically-informed studies of adolescence are an absolute necessity if we are to improve the mental health and well-being of future generations. Vital initiatives such as NIH’s ABCD study tracking the maturation of ~12,000 10-year-olds are poised to make critical contributions to our understanding of how adolescent brains create adult minds. Based on the evidence presented in this review, it would be highly beneficial if these efforts extend beyond subjective questionnaires and pay detailed attention to sleep and sleep-dependent neural network activity.

Finally, promoting lifestyle choices that support healthy adolescent sleep, including exercise, later school start times and reduced mobile phone and internet usage, may bring significant benefits. Such societal shifts may facilitate the successful transition from adolescent to adult, permitting the insights and benefits gained from good quality sleep – “nature’s soft nurse” (Shakespeare, Henry IV pt 2) – to last a lifetime.

## Supplementary Material

Appendix

## References

[R1] Alvaro PK, Roberts RM, Harris JK (2014). The independent relationships between insomnia, depression, subtypes of anxiety, and chronotype during adolescence. Sleep Med.

[R2] Anastasiades PG, Marques-Smith A, Lyngholm D, Lickiss T, Raffiq S, Kätzel D, Miesenböck G, Butt SJB (2016). GABAergic interneurons form transient layer-specific circuits in early postnatal neocortex. Nat Commun.

[R3] Anderson SA, Classey JD, Condé F, Lund JS, Lewis DA (1995). Synchronous development of pyramidal neuron dendritic spines and parvalbumin-immunoreactive chandelier neuron axon terminals in layer III of monkey prefrontal cortex. Neuroscience.

[R4] Andorko ND, Mittal V, Thompson E, Denenny D, Epstein G, Demro C, Wilson C, Sun S, Klingaman EA, DeVylder J, Oh H (2017). The association between sleep dysfunction and psychosis-like experiences among college students. Psychiatry Res.

[R5] Andreae LC, Burrone J (2014). The role of neuronal activity and transmitter release on synapse formation. Curr Opin Neurobiol.

[R6] Arnsten AFT, Girgis RR, Gray DL, Mailman RB (2017). Novel Dopamine Therapeutics for Cognitive Deficits in Schizophrenia. Biol Psychiatry.

[R7] Arruda-Carvalho M, Wu W-C, Cummings KA, Clem RL (2017). Optogenetic Examination of Prefrontal-Amygdala Synaptic Development. J Neurosci.

[R8] Aton SJ, Seibt J, Dumoulin M, Jha SK, Steinmetz N, Coleman T, Naidoo N, Frank MG (2009). Mechanisms of sleep-dependent consolidation of cortical plasticity. Neuron.

[R9] Baglioni C, Battagliese G, Feige B, Spiegelhalder K, Nissen C, Voderholzer U, Lombardo C, Riemann D (2011). Insomnia as a predictor of depression: A meta-analytic evaluation of longitudinal epidemiological studies. Journal of Affective Disorders.

[R10] Banks S, Van Dongen HPA, Maislin G, Dinges DF (2010). Neurobehavioral Dynamics Following Chronic Sleep Restriction: Dose-Response Effects of One Night for Recovery. Sleep.

[R11] Basner M, Rao H, Goel N, Dinges DF (2013). Sleep deprivation and neurobehavioral dynamics. Current Opinion in Neurobiology, Circadian rhythm and sleep.

[R12] Bayless DW, Daniel JM (2015). Sex differences in myelin-associated protein levels within and density of projections between the orbital frontal cortex and dorsal striatum of adult rats: Implications for inhibitory control. Neuroscience.

[R13] Bayless DW, Darling JS, Daniel JM (2013). Mechanisms by which neonatal testosterone exposure mediates sex differences in impulsivity in prepubertal rats. Hormones and Behavior.

[R14] Beebe DW, DiFrancesco MW, Tlustos SJ, McNally KA, Holland SK (2009). Preliminary fMRI findings in experimentally sleep-restricted adolescents engaged in a working memory task. Behav Brain Funct.

[R15] Beebe DW, Field J, Milller MM, Miller LE, LeBlond E (2017). Impact of Multi-Night Experimentally Induced Short Sleep on Adolescent Performance in a Simulated Classroom. Sleep.

[R16] Belenky G, Wesensten NJ, Thorne DR, Thomas ML, Sing HC, Redmond DP, Russo MB, Balkin TJ (2003). Patterns of performance degradation and restoration during sleep restriction and subsequent recovery: a sleep dose-response study. Journal of Sleep Research.

[R17] Bellesi M, de Vivo L (2020). Structural synaptic plasticity across sleep and wake. Current Opinion in Physiology, Physiology of sleep.

[R18] Bellesi M, de Vivo L, Chini M, Gilli F, Tononi G, Cirelli C (2017). Sleep Loss Promotes Astrocytic Phagocytosis and Microglial Activation in Mouse Cerebral Cortex. J Neurosci.

[R19] Bellesi M, de Vivo L, Tononi G, Cirelli C (2015). Effects of sleep and wake on astrocytes: clues from molecular and ultrastructural studies. BMC Biol.

[R20] Bellesi M, Haswell JD, de Vivo L, Marshall W, Roseboom PH, Tononi G, Cirelli C (2018). Myelin modifications after chronic sleep loss in adolescent mice. Sleep.

[R21] Bellesi M, Pfister-Genskow M, Maret S, Keles S, Tononi G, Cirelli C (2013). Effects of Sleep and Wake on Oligodendrocytes and Their Precursors. J Neurosci.

[R22] Bellesi M, Tononi G, Cirelli C, Serra PA (2016). Region-Specific Dissociation between Cortical Noradrenaline Levels and the Sleep/Wake Cycle. Sleep.

[R23] Benes FM (1989). Myelination of Cortical-hippocampal Relays During Late Adolescence. Schizophrenia Bulletin.

[R24] Berman S, West KL, Does MD, Yeatman JD, Mezer AA (2018). Evaluating g-ratio weighted changes in the corpus callosum as a function of age and sex. NeuroImage, Microstructural Imaging.

[R25] Bernstein H-G, Steiner J, Guest PC, Dobrowolny H, Bogerts B (2015). Glial cells as key players in schizophrenia pathology: recent insights and concepts of therapy. Schizophrenia Research, White Matter Pathology.

[R26] Bhattacherjee A, Djekidel MN, Chen R, Chen W, Tuesta LM, Zhang Y (2019). Cell type-specific transcriptional programs in mouse prefrontal cortex during adolescence and addiction. Nat Commun.

[R27] Bicks LK, Yamamuro K, Flanigan ME, Kim JM, Kato D, Lucas EK, Koike H, Peng MS, Brady DM, Chandrasekaran S, Norman KJ (2020). Prefrontal parvalbumin interneurons require juvenile social experience to establish adult social behavior. Nat Commun.

[R28] Billeh YN, Rodriguez AV, Bellesi M, Bernard A, de Vivo L, Funk CM, Harris J, Honjoh S, Mihalas S, Ng L, Koch C (2016). Effects of Chronic Sleep Restriction during Early Adolescence on the Adult Pattern of Connectivity of Mouse Secondary Motor Cortex. eNeuro.

[R29] Bitzenhofer SH, Pöpplau JA, Chini M, Marquardt A, Hanganu-Opatz IL (2021). A transient developmental increase in prefrontal activity alters network maturation and causes cognitive dysfunction in adult mice. Neuron.

[R30] Blakemore S-J (2019). Adolescence and mental health. The Lancet.

[R31] Blakemore S-J, Mills KL (2014). Is Adolescence a Sensitive Period for Sociocultural Processing?. Annual Review of Psychology.

[R32] van den Bos W, Rodriguez CA, Schweitzer JB, McClure SM (2015). Adolescent impatience decreases with increased frontostriatal connectivity. PNAS.

[R33] Bourgeois J-P, Goldman-Rakic PS, Rakic P (1994). Synaptogenesis in the Prefrontal Cortex of Rhesus Monkeys. Cerebral Cortex.

[R34] Breslau N, Roth T, Rosenthal L, Andreski P (1996). Sleep disturbance and psychiatric disorders: A longitudinal epidemiological study of young Adults. Biological Psychiatry.

[R35] Bridi MCD, Zong F-J, Min X, Luo N, Tran T, Qiu J, Severin D, Zhang X-T, Wang G, Zhu Z-J, He K-W (2019). Daily Oscillation of the Excitation-Inhibition Balance in Visual Cortical Circuits. Neuron.

[R36] Brooks SJ, Katz ES, Stamoulis C (2022). Shorter Duration and Lower Quality Sleep Have Widespread Detrimental Effects on Developing Functional Brain Networks in Early Adolescence. Cerebral Cortex Communications.

[R37] Bruni O, Giallonardo M, Sacco R, Ferri R, Melegari MG (2021). The impact of lockdown on sleep patterns of children and adolescents with ADHD. Journal of Clinical Sleep Medicine.

[R38] Brüning F, Noya SB, Bange T, Koutsouli S, Rudolph JD, Tyagarajan SK, Cox J, Mann M, Brown SA, Robles MS (2019). Sleep-wake cycles drive daily dynamics of synaptic phosphorylation. Science.

[R39] Caballero A, Flores-Barrera E, Thomases DR, Tseng KY (2020). Downregulation of parvalbumin expression in the prefrontal cortex during adolescence causes enduring prefrontal disinhibition in adulthood. Neuropsychopharmacol.

[R40] Caballero A, Tseng KY (2016). GABAergic Function as a Limiting Factor for Prefrontal Maturation during Adolescence. Trends Neurosci.

[R41] Camchong J, Lim KO, Sponheim SR, MacDonald AW (2009). Frontal white matter integrity as an endophenotype for schizophrenia: diffusion tensor imaging in monozygotic twins and patients’ nonpsychotic relatives. Front Hum Neurosci.

[R42] Canetta SE, Holt ES, Benoit LJ, Teboul E, Ogden RT, Harris AZ, Kellendonk C (2021). Mature parvalbumin interneuron function in prefrontal cortex requires activity during a postnatal sensitive period. bioRxiv.

[R43] Carlén M, Meletis K, Siegle JH, Cardin JA, Futai K, Vierling-Claassen D, Rühlmann C, Jones SR, Deisseroth K, Sheng M, Moore CI (2012). A critical role for NMDA receptors in parvalbumin interneurons for gamma rhythm induction and behavior. Mol Psychiatry.

[R44] Carskadon MA (2011). Sleep in Adolescents: The Perfect Storm. Pediatr Clin North Am.

[R45] Cercignani M, Giulietti G, Dowell NG, Gabel M, Broad R, Leigh PN, Harrison NA, Bozzali M (2017). Characterizing axonal myelination within the healthy population: a tract-by-tract mapping of effects of age and gender on the fiber g-ratio. Neurobiology of Aging.

[R46] Cheng W, Rolls E, Gong W, Du J, Zhang J, Zhang X-Y, Li F, Feng J (2020). Sleep duration, brain structure, and psychiatric and cognitive problems in children. Mol Psychiatry.

[R47] Chung W-S, Clarke LE, Wang GX, Stafford BK, Sher A, Chakraborty C, Joung J, Foo LC, Thompson A, Chen C, Smith SJ (2013). Astrocytes mediate synapse elimination through MEGF10 and MERTK pathways. Nature.

[R48] Cirelli C, Gutierrez CM, Tononi G (2004). Extensive and divergent effects of sleep and wakefulness on brain gene expression. Neuron.

[R49] Clarke G, Harvey AG (2012). The Complex Role of Sleep in Adolescent Depression. Child Adolesc Psychiatr Clin N Am.

[R50] Clemens AM, Lenschow C, Beed P, Li L, Sammons R, Naumann RK, Wang H, Schmitz D, Brecht M (2019). Estrus-Cycle Regulation of Cortical Inhibition. Current Biology.

[R51] Counotte DS, Li KW, Wortel J, Gouwenberg Y, Schors RCVD, Smit AB, Spijker S (2010). Changes in molecular composition of rat medial prefrontal cortex synapses during adolescent development. European Journal of Neuroscience.

[R52] Crone EA, Konijn EA (2018). Media use and brain development during adolescence. Nature Communications.

[R53] Crouse JJ, Carpenter JS, Song YJC, Hockey SJ, Naismith SL, Grunstein RR, Scott EM, Merikangas KR, Scott J, Hickie IB (2021). Circadian rhythm sleep-wake disturbances and depression in young people: implications for prevention and early intervention. Lancet Psychiatry.

[R54] Cui J, Tkachenko O, Gogel H, Kipman M, Preer LA, Weber M, Divatia SC, Demers LA, Olson EA, Buchholz JL, Bark JS (2015). Microstructure of frontoparietal connections predicts individual resistance to sleep deprivation. NeuroImage.

[R55] Cunningham JEA, Shapiro CM (2018). Cognitive Behavioural Therapy for Insomnia (CBT-I) to treat depression: A systematic review. Journal of Psychosomatic Research.

[R56] da Silva Rocha-Lopes J, Machado RB, Suchecki D (2018). Chronic REM Sleep Restriction in Juvenile Male Rats Induces Anxiety-Like Behavior and Alters Monoamine Systems in the Amygdala and Hippocampus. Mol Neurobiol.

[R57] Darling JS, Bayless DW, Dartez LR, Taylor JJ, Mehrotra A, Smith WL, Daniel JM (2020). Sex differences in impulsivity in adult rats are mediated by organizational actions of neonatal gonadal hormones and not by hormones acting at puberty or in adulthood. Behav Brain Res.

[R58] Dauvilliers Y, Maret S, Tafti M (2005). Genetics of normal and pathological sleep in humans. Sleep Medicine Reviews.

[R59] Davies G, Haddock G, Yung AR, Mulligan LD, Kyle SD (2017). A systematic review of the nature and correlates of sleep disturbance in early psychosis. Sleep Med Rev.

[R60] Davla S, Artiushin G, Li Y, Chitsaz D, Li S, Sehgal A, van Meyel DJ (2020). AANAT1 functions in astrocytes to regulate sleep homeostasis. eLife.

[R61] De Havas JA, Parimal S, Soon CS, Chee MWL (2012). Sleep deprivation reduces default mode network connectivity and anti-correlation during rest and task performance. NeuroImage.

[R62] de Vivo L, Bellesi M, Marshall W, Bushong EA, Ellisman MH, Tononi G, Cirelli C (2017). Ultrastructural evidence for synaptic scaling across the wake/sleep cycle. Science.

[R63] de Vivo L, Nagai H, De Wispelaere N, Spano GM, Marshall W, Bellesi M, Nemec KM, Schiereck SS, Nagai M, Tononi G, Cirelli C (2019). Evidence for sleep-dependent synaptic renormalization in mouse pups. Sleep.

[R64] Dienel SJ, Lewis DA (2018). Alterations in cortical interneurons and cognitive function in schizophrenia. Neurobiol Dis.

[R65] Diering GH, Nirujogi RS, Roth RH, Worley PF, Pandey A, Huganir RL (2017). Homer1a drives homeostatic scaling-down of excitatory synapses during sleep. Science.

[R66] Dorsey A, de Lecea L, Jennings KJ (2021). Neurobiological and Hormonal Mechanisms Regulating Women’s Sleep. Frontiers in Neuroscience.

[R67] Drzewiecki CM, Willing J, Juraska JM (2016). Synaptic number changes in the medial prefrontal cortex across adolescence in male and female rats: A role for pubertal onset. Synapse.

[R68] Ehlert U (2013). Enduring psychobiological effects of childhood adversity. Psychoneuroendocrinology.

[R69] Elvsåshagen T, Norbom LB, Pedersen PØ, Quraishi SH, Bjørnerud A, Malt UF, Groote IR, Westlye LT (2015). Widespread changes in white matter microstructure after a day of waking and sleep deprivation. PLoS ONE.

[R70] Facer-Childs ER, Campos BM, Middleton B, Skene DJ, Bagshaw AP (2019). Circadian phenotype impacts the brain’s resting-state functional connectivity, attentional performance, and sleepiness. Sleep.

[R71] Fair DA, Cohen AL, Power JD, Dosenbach NUF, Church JA, Miezin FM, Schlaggar BL, Petersen SE (2009). Functional Brain Networks Develop from a “Local to Distributed” Organization. PLoS Comput Biol.

[R72] Fogaça MV, Duman RS (2019). Cortical GABAergic Dysfunction in Stress and Depression: New Insights for Therapeutic Interventions. Front Cell Neurosci.

[R73] Frank MG (2017). Sleep and plasticity in the visual cortex: More than meets the eye. Curr Opin Neurobiol.

[R74] Freeman D, Sheaves B, Goodwin GM, Yu L-M, Nickless A, Harrison PJ, Emsley R, Luik AI, Foster RG, Wadekar V, Hinds C (2017). The effects of improving sleep on mental health (OASIS): a randomised controlled trial with mediation analysis. Lancet Psychiatry.

[R75] Freeman D, Waite F, Startup H, Myers E, Lister R, McInerney J, Harvey AG, Geddes J, Zaiwalla Z, Luengo-Fernandez R, Foster R (2015). Efficacy of cognitive behavioural therapy for sleep improvement in patients with persistent delusions and hallucinations (BEST): a prospective, assessor-blind, randomised controlled pilot trial. Lancet Psychiatry.

[R76] Fuligni AJ, Hardway C (2006). Daily Variation in Adolescents’ Sleep, Activities, and Psychological Well-Being. Journal of Research on Adolescence.

[R77] Galván A (2020). The Need for Sleep in the Adolescent Brain. Trends in Cognitive Sciences.

[R78] Gangwisch JE, Babiss LA, Malaspina D, Turner JB, Zammit GK, Posner K (2010). Earlier Parental Set Bedtimes as a Protective Factor Against Depression and Suicidal Ideation. Sleep.

[R79] Gee DG, Humphreys KL, Flannery J, Goff B, Telzer EH, Shapiro M, Hare TA, Bookheimer SY, Tottenham N (2013). A Developmental Shift from Positive to Negative Connectivity in Human Amygdala–Prefrontal Circuitry. J Neurosci.

[R80] Glausier JR, Lewis DA (2013). Dendritic Spine Pathology in Schizophrenia. Neuroscience.

[R81] Gobin CM, Banks JB, Fins AI, Tartar JL (2015). Poor sleep quality is associated with a negative cognitive bias and decreased sustained attention. Journal of Sleep Research.

[R82] Gogtay N, Giedd JN, Lusk L, Hayashi KM, Greenstein D, Vaituzis AC, Nugent TF, Herman DH, Clasen LS, Toga AW, Rapoport JL (2004). Dynamic mapping of human cortical development during childhood through early adulthood. Proc Natl Acad Sci USA.

[R83] Goldstein AN, Walker MP (2014). The role of sleep in emotional brain function. Annu Rev Clin Psychol.

[R84] Goldstone A, Javitz HS, Claudatos SA, Buysse DJ, Hasler BP, de Zambotti M, Clark DB, Franzen PL, Prouty DE, Colrain IM, Baker FC (2020). Sleep Disturbance Predicts Depression Symptoms in Early Adolescence: Initial Findings From the Adolescent Brain Cognitive Development Study. Journal of Adolescent Health.

[R85] Gomes FV, Grace AA (2017). Prefrontal Cortex Dysfunction Increases Susceptibility to Schizophrenia-Like Changes Induced by Adolescent Stress Exposure. Schizophrenia Bulletin.

[R86] Gonzalez-Burgos G, Miyamae T, Pafundo DE, Yoshino H, Rotaru DC, Hoftman G, Datta D, Zhang Y, Hammond M, Sampson AR, Fish KN (2015). Functional Maturation of GABA Synapses During Postnatal Development of the Monkey Dorsolateral Prefrontal Cortex. Cereb Cortex.

[R87] González-Rueda A, Pedrosa V, Feord RC, Clopath C, Paulsen O (2018). Activity-Dependent Downscaling of Subthreshold Synaptic Inputs during Slow-Wave-Sleep-like Activity In Vivo. Neuron.

[R88] Gruber R, Gauthier-Gagne G, Voutou D, Somerville G, Saha S, Boursier J (2021). Pre-pandemic sleep behavior and adolescents’ stress during Covid-19: a prospective longitudinal study. Child and Adolescent Psychiatry and Mental Health.

[R89] Gruber R, Saha S, Somerville G, Boursier J, Wise MS (2020). The impact of COVID-19 related school shutdown on sleep in adolescents: a natural experiment. Sleep Medicine.

[R90] Harkness JH, Bushana PN, Todd RP, Clegern WC, Sorg BA, Wisor JP (2019). Sleep disruption elevates oxidative stress in parvalbumin-positive cells of the rat cerebral cortex. Sleep.

[R91] Harvey AG (2016). A Transdiagnostic Intervention for Youth Sleep and Circadian Problems. Cognitive and Behavioral Practice, Emerging Adulthood: Developmental and Clinical Considerations in Developing Efficacious Interventions for College-Aged Populations.

[R92] Hashemi E, Ariza J, Rogers H, Noctor SC, Martínez-Cerdeño V (2017). The Number of Parvalbumin-Expressing Interneurons Is Decreased in the Prefrontal Cortex in Autism. Cereb Cortex.

[R93] Hashimoto T, Nguyen QL, Rotaru D, Keenan T, Arion D, Beneyto M, Gonzalez-Burgos G, Lewis DA (2009). Protracted Developmental Trajectories of GABAA Receptor α1 and α2 Subunit Expression in Primate Prefrontal Cortex. Biol Psychiatry.

[R94] Haydon PG (2017). Astrocytes and the Modulation of Sleep. Curr Opin Neurobiol.

[R95] Heissel JA, Sharkey PT, Torrats-Espinosa G, Grant K, Adam EK (2018). Violence and Vigilance: The Acute Effects of Community Violent Crime on Sleep and Cortisol. Child Development.

[R96] Hennig T, Lincoln TM (2018). Sleeping Paranoia Away? An Actigraphy and Experience-Sampling Study with Adolescents. Child Psychiatry Hum Dev.

[R97] Holmes SE, Scheinost D, Finnema SJ, Naganawa M, Davis MT, DellaGioia N, Nabulsi N, Matuskey D, Angarita GA, Pietrzak RH, Duman RS (2019). Lower synaptic density is associated with depression severity and network alterations. Nat Commun.

[R98] Homberg JR, Adan RAH, Alenina N, Asiminas A, Bader M, Beckers T, Begg DP, Blokland A, Burger ME, van Dijk G, Eisel ULM (2021). The continued need for animals to advance brain research. Neuron.

[R99] Hysing M, Harvey AG, Linton SJ, Askeland KG, Sivertsen B (2016). Sleep and academic performance in later adolescence: results from a large population-based study. Journal of Sleep Research.

[R100] Imtiaz SA (2021). A Systematic Review of Sensing Technologies for Wearable Sleep Staging. Sensors.

[R101] Emslie J, Armitage R, Weinberg WA, Rush AJ, Mayes TL, Hoffmann RF (2001). Sleep polysomnography as a predictor of recurrence in children and adolescents with major depressive disorder. International Journal of Neuropsychopharmacology.

[R102] Jalbrzikowski M, Hayes RA, Scully KE, Franzen PL, Hasler BP, Siegle GJ, Buysse DJ, Dahl RE, Forbes EE, Ladouceur CD, McMakin DL (2021). Associations between brain structure and sleep patterns across adolescent development. Sleep.

[R103] Jarrin DC, McGrath JJ, Quon EC (2014). Objective and Subjective Socioeconomic Gradients Exist for Sleep in Children and Adolescents. Health Psychol.

[R104] Joffe ME, Santiago CI, Oliver KH, Maksymetz J, Harris NA, Engers JL, Lindsley CW, Winder DG, Conn PJ (2020). mGlu2 and mGlu3 Negative Allosteric Modulators Divergently Enhance Thalamocortical Transmission and Exert Rapid Antidepressant-like Effects. Neuron.

[R105] Jones CE, Opel RA, Kaiser ME, Chau AQ, Quintana JR, Nipper MA, Finn DA, Hammock EAD, Lim MM (2019). Early-life sleep disruption increases parvalbumin in primary somatosensory cortex and impairs social bonding in prairie voles. Science Advances.

[R106] Kahn-Greene ET, Killgore DB, Kamimori GH, Balkin TJ, Killgore WDS (2007). The effects of sleep deprivation on symptoms of psychopathology in healthy adults. Sleep Med.

[R107] Kang HJ, Voleti B, Hajszan T, Rajkowska G, Stockmeier C, Licznerski P, Lepack A, Majik MS, Jeong LS, Banasr M, Son H (2012). Decreased Expression of Synapse-Related Genes and Loss of Synapses in Major Depressive Disorder. Nat Med.

[R108] Keyes KM, Maslowsky J, Hamilton A, Schulenberg J (2015). The great sleep recession: changes in sleep duration among US adolescents, 1991-2012. Pediatrics.

[R109] Killgore WDS (2013). Self-Reported Sleep Correlates with Prefrontal-Amygdala Functional Connectivity and Emotional Functioning. Sleep.

[R110] Kim E-J, Dimsdale JE (2007). The Effect of Psychosocial Stress on Sleep: A Review of Polysomnographic Evidence. Behav Sleep Med.

[R111] Knudsen EI (2004). Sensitive periods in the development of the brain and behavior. J Cogn Neurosci.

[R112] Koss WA, Belden CE, Hristov AD, Juraska JM (2014). Dendritic remodeling in the adolescent medial prefrontal cortex and the basolateral amygdala of male and female rats. Synapse.

[R113] Koyanagi A, Stickley A (2015). The Association between Sleep Problems and Psychotic Symptoms in the General Population: A Global Perspective. Sleep.

[R114] Kraepelin E (1919). Dementia praecox and paraphrenia.

[R115] Krause AJ, Ben Simon E, Mander BA, Greer SM, Saletin JM, Goldstein-Piekarski AN, Walker MP (2017). The sleep-deprived human brain. Nat Rev Neurosci.

[R116] Kurth S, Ringli M, Geiger A, LeBourgeois M, Jenni OG, Huber R (2010). Mapping of cortical activity in the first two decades of life: a high-density sleep electroencephalogram study. J Neurosci.

[R117] Larsen B, Luna B (2018). Adolescence as a neurobiological critical period for the development of higher-order cognition. Neurosci Biobehav Rev.

[R118] Laubach M, Amarante LM, Swanson K, White SR (2018). What, If Anything, Is Rodent Prefrontal Cortex?. eNeuro.

[R119] Laviola G, Macrí S, Morley-Fletcher S, Adriani W (2003). Risk-taking behavior in adolescent mice: psychobiological determinants and early epigenetic influence. Neuroscience & Biobehavioral Reviews, Brain Development, Sex Differences and Stress: Implications for Psychopathology.

[R120] Lee YJ, Cho S-J, Cho IH, Jang JH, Kim S-J (2012a). The relationship between psychotic-like experiences and sleep disturbances in adolescents. Sleep Medicine.

[R121] Lee YJ, Cho SJ, Cho IH, Kim SJ (2012b). Insufficient Sleep and Suicidality in Adolescents. Sleep.

[R122] Lemola S, Ledermann T, Friedman EM (2013). Variability of Sleep Duration Is Related to Subjective Sleep Quality and Subjective Well-Being: An Actigraphy Study. PLOS ONE.

[R123] Lenroot RK, Gogtay N, Greenstein DK, Wells EM, Wallace GL, Clasen LS, Blumenthal JD, Lerch J, Zijdenbos AP, Evans AC, Thompson PM (2007). Sexual Dimorphism of Brain Developmental Trajectories during Childhood and Adolescence. Neuroimage.

[R124] Lewis DA (1997). Development of the Prefrontal Cortex during Adolescence: Insights into Vulnerable Neural Circuits in Schizophrenia. Neuropsychopharmacol.

[R125] Li W, Ma L, Yang G, Gan WB (2017). REM sleep selectively prunes and maintains new synapses in development and learning. Nat Neurosci.

[R126] Lian Q, Zuo X, Zhong X, Tu X, Zhang J, Shu C, Yu C, Lou C (2021). The effect of COVID-19 school closures on adolescent sleep duration: an uncontrolled before-after study. BMC Public Health.

[R127] Liston C, Watts R, Tottenham N, Davidson MC, Niogi S, Ulug AM, Casey BJ (2006). Frontostriatal Microstructure Modulates Efficient Recruitment of Cognitive Control. Cerebral Cortex.

[R128] Liu X (2004). Sleep and Adolescent Suicidal Behavior. Sleep.

[R129] Liu Z, Wang Y, Cai L, Li Y, Chen B, Dong Y, Huang YH (2016). Prefrontal Cortex to Accumbens Projections in Sleep Regulation of Reward. J Neurosci.

[R130] Lo JC, Chee MW (2020). Cognitive effects of multi-night adolescent sleep restriction: current data and future possibilities. Current Opinion in Behavioral Sciences, Sleep and cognition.

[R131] Logan RW, Hasler BP, Forbes EE, Franzen PL, Torregrossa MM, Huang YH, Buysse DJ, Clark DB, McClung CA (2018). Impact of Sleep and Circadian Rhythms on Addiction Vulnerability in Adolescents. Biological Psychiatry, Impulsivity: Mechanisms and Manifestations.

[R132] Lovato N, Short MA, Micic G, Hiller RM, Gradisar M (2017). An investigation of the longitudinal relationship between sleep and depressed mood in developing teens. Nat Sci Sleep.

[R133] Luna B, Thulborn KR, Munoz DP, Merriam EP, Garver KE, Minshew NJ, Keshavan MS, Genovese CR, Eddy WF, Sweeney JA (2001). Maturation of Widely Distributed Brain Function Subserves Cognitive Development. NeuroImage.

[R134] Lunsford-Avery JR, Damme KSF, Engelhard MM, Kollins SH, Mittal VA (2020). Sleep/Wake Regularity Associated with Default Mode Network Structure among Healthy Adolescents and Young Adults. Sci Rep.

[R135] Lunsford-Avery JR, Gonçalves B da SB, Brietzke E, Bressan RA, Gadelha A, Auerbach RP, Mittal VA (2017). Adolescents at clinical-high risk for psychosis: Circadian rhythm disturbances predict worsened prognosis at 1-year follow-up. Schizophr Res.

[R136] Lunsford-Avery JR, Orr JM, Gupta T, Pelletier-Baldelli A, Dean DJ, Smith Watts AK, Bernard J, Millman ZB, Mittal VA (2013). Sleep dysfunction and thalamic abnormalities in adolescents at ultra high-risk for psychosis. Schizophr Res.

[R137] Luscher B, Shen Q, Sahir N (2011). The GABAergic Deficit Hypothesis of Major Depressive Disorder. Mol Psychiatry.

[R138] Maas DA, Vallès A, Martens GJM (2017). Oxidative stress, prefrontal cortex hypomyelination and cognitive symptoms in schizophrenia. Transl Psychiatry.

[R139] Makinodan M, Rosen KM, Ito S, Corfas G (2012). A Critical Period for Social Experience–Dependent Oligodendrocyte Maturation and Myelination. Science.

[R140] Mallya AP, Wang H-D, Lee HNR, Deutch AY (2019). Microglial Pruning of Synapses in the Prefrontal Cortex During Adolescence. Cerebral Cortex.

[R141] Maret S, Faraguna U, Nelson AB, Cirelli C, Tononi G (2011). Sleep and waking modulate spine turnover in the adolescent mouse cortex. Nat Neurosci.

[R142] Markovic A, Kaess M, Tarokh L (2020). Environmental Factors Shape Sleep EEG Connectivity During Early Adolescence. Cerebral Cortex.

[R143] Mason MF, Norton MI, Van Horn JD, Wegner DM, Grafton ST, Macrae CN (2007). Wandering minds: the default network and stimulus-independent thought. Science.

[R144] Mayeli A, LaGoy A, Donati FL, Kaskie RE, Najibi SM, Ferrarelli F (2021). Sleep abnormalities in individuals at clinical high risk for psychosis. J Psychiatr Res.

[R145] McCormick CM, Mathews IZ (2007). HPA function in adolescence: role of sex hormones in its regulation and the enduring consequences of exposure to stressors. Pharmacol Biochem Behav.

[R146] Miller EK, Cohen JD (2001). An integrative theory of prefrontal cortex function. Annu Rev Neurosci.

[R147] Miller GE, Chen E, Armstrong CC, Carroll AL, Ozturk S, Rydland KJ, Brody GH, Parrish TB, Nusslock R (2018). Functional connectivity in central executive network protects youth against cardiometabolic risks linked with neighborhood violence. PNAS.

[R148] Miyawaki T, Norimoto H, Ishikawa T, Watanabe Y, Matsuki N, Ikegaya Y (2014). Dopamine receptor activation reorganizes neuronal ensembles during hippocampal sharp waves in vitro. PLoS ONE.

[R149] Mongrain V, Hernandez SA, Pradervand S, Dorsaz S, Curie T, Hagiwara G, Gip P, Heller HC, Franken P (2010). Separating the contribution of glucocorticoids and wakefulness to the molecular and electrophysiological correlates of sleep homeostasis. Sleep.

[R150] Mulligan LD, Haddock G, Emsley R, Neil ST, Kyle SD (2016). High resolution examination of the role of sleep disturbance in predicting functioning and psychotic symptoms in schizophrenia: A novel experience sampling study. J Abnorm Psychol.

[R151] Murphy M, Riedner BA, Huber R, Massimini M, Ferrarelli F, Tononi G (2009). Source modeling sleep slow waves. PNAS.

[R152] Nabel EM, Garkun Y, Koike H, Sadahiro M, Liang A, Norman KJ, Taccheri G, Demars MP, Im S, Caro K, Lopez S (2020). Adolescent frontal top-down neurons receive heightened local drive to establish adult attentional behavior in mice. Nat Commun.

[R153] Nagy Z, Westerberg H, Klingberg T (2004). Maturation of White Matter is Associated with the Development of Cognitive Functions during Childhood. Journal of Cognitive Neuroscience.

[R154] Najjar S, Pearlman DM, Alper K, Najjar A, Devinsky O (2013). Neuroinflammation and psychiatric illness. Journal of Neuroinflammation.

[R155] O’Callaghan VS, Couvy-Duchesne B, Strike LT, McMahon KL, Byrne EM, Wright MJ (2021). A meta-analysis of the relationship between subjective sleep and depressive symptoms in adolescence. Sleep Medicine.

[R156] O’Connell KS, Frei O, Bahrami S, Smeland OB, Bettella F, Cheng W, Chu Y, Hindley G, Lin A, Shadrin A, Barrett EA (2021). Characterizing the Genetic Overlap Between Psychiatric Disorders and Sleep-Related Phenotypes. Biological Psychiatry, Rare and Common Genetic Variance and Psychosis.

[R157] Ojio Y, Nishida A, Shimodera S, Togo F, Sasaki T (2016). Sleep Duration Associated with the Lowest Risk of Depression/Anxiety in Adolescents. Sleep.

[R158] Orchard F, Gregory AM, Gradisar M, Reynolds S (2020). Self-reported sleep patterns and quality amongst adolescents: cross-sectional and prospective associations with anxiety and depression. Journal of Child Psychology and Psychiatry.

[R159] Oshima N, Nishida A, Fukushima M, Shimodera S, Kasai K, Okazaki Y, Sasaki T (2010). Psychotic-like experiences (PLEs) and mental health status in twin and singleton Japanese high school students. Early Intervention in Psychiatry.

[R160] Owens J, Group ASW, Adolescence CO (2014). Insufficient Sleep in Adolescents and Young Adults: An Update on Causes and Consequences. Pediatrics peds.

[R161] Pacheco AT, Bottorff J, Gao Y, Turrigiano GG (2021). Sleep Promotes Downward Firing Rate Homeostasis. Neuron.

[R162] Paolicelli RC, Bolasco G, Pagani F, Maggi L, Scianni M, Panzanelli P, Giustetto M, Ferreira TA, Guiducci E, Dumas L, Ragozzino D (2011). Synaptic Pruning by Microglia Is Necessary for Normal Brain Development. Science.

[R163] Pasch KE, Latimer LA, Cance JD, Moe SG, Lytle LA (2012). Longitudinal Bi-directional Relationships Between Sleep and Youth Substance Use. J Youth Adolesc.

[R164] Pattwell SS, Liston C, Jing D, Ninan I, Yang RR, Witztum J, Murdock MH, Dincheva I, Bath KG, Casey BJ, Deisseroth K (2016). Dynamic changes in neural circuitry during adolescence are associated with persistent attenuation of fear memories. Nat Commun.

[R165] Paus T, Keshavan M, Giedd JN (2008). Why do many psychiatric disorders emerge during adolescence?. Nat Rev Neurosci.

[R166] Peach H, Gaultney JF, Gray DD (2016). Sleep hygiene and sleep quality as predictors of positive and negative dimensions of mental health in college students. Cogent Psychology.

[R167] Perova Z, Delevich K, Li B (2015). Depression of Excitatory Synapses onto Parvalbumin Interneurons in the Medial Prefrontal Cortex in Susceptibility to Stress. J Neurosci.

[R168] Perrin JS, Hervé P-Y, Leonard G, Perron M, Pike GB, Pitiot A, Richer L, Veillette S, Pausova Z, Paus T (2008). Growth of White Matter in the Adolescent Brain: Role of Testosterone and Androgen Receptor. J Neurosci.

[R169] Petanjek Z, Judaš M, Šimic G, Rasin MR, Uylings HBM, Rakic P, Kostovic I (2011). Extraordinary neoteny of synaptic spines in the human prefrontal cortex. Proc Natl Acad Sci USA.

[R170] Philip P, Sagaspe P, Prague M, Tassi P, Capelli A, Bioulac B, Commenges D, Taillard J (2012). Acute Versus Chronic Partial Sleep Deprivation in Middle-Aged People: Differential Effect on Performance and Sleepiness. Sleep.

[R171] Piekarski DJ, Boivin JR, Wilbrecht L (2017). Ovarian Hormones Organize the Maturation of Inhibitory Neurotransmission in the Frontal Cortex at Puberty Onset in Female Mice. Curr Biol.

[R172] Pilcher JJ, Ginter DR, Sadowsky B (1997). Sleep quality versus sleep quantity: Relationships between sleep and measures of health, well-being and sleepiness in college students. Journal of Psychosomatic Research, Nocturnal Penile Tumescence: Measurement and Reasearch.

[R173] Preuss TM, Wise SP (2021). Evolution of prefrontal cortex. Neuropsychopharmacol.

[R174] Rajkowska G, Miguel-Hidalgo JJ, Makkos Z, Meltzer H, Overholser J, Stockmeier C (2002). Layer-specific reductions in GFAP-reactive astroglia in the dorsolateral prefrontal cortex in schizophrenia. Schizophrenia Research.

[R175] Raznahan A, Lee Y, Stidd R, Long R, Greenstein D, Clasen L, Addington A, Gogtay N, Rapoport JL, Giedd JN (2010). Longitudinally mapping the influence of sex and androgen signaling on the dynamics of human cortical maturation in adolescence. Proc Natl Acad Sci U S A.

[R176] Raznahan A, Lerch JP, Lee N, Greenstein D, Wallace GL, Stockman M, Clasen L, Shaw PW, Giedd JN (2011). Patterns of Coordinated Anatomical Change in Human Cortical Development: A Longitudinal Neuroimaging Study of Maturational Coupling. Neuron.

[R177] Reeve S, Emsley R, Sheaves B, Freeman D (2018). Disrupting Sleep: The Effects of Sleep Loss on Psychotic Experiences Tested in an Experimental Study With Mediation Analysis. Schizophr Bull.

[R178] Reeve S, Nickless A, Sheaves B, Hodgekins J, Stewart SLK, Gumley A, Fowler D, Morrison A, Freeman D (2019). Sleep duration and psychotic experiences in patients at risk of psychosis: A secondary analysis of the EDIE-2 trial. Schizophr Res.

[R179] Sarah Reeve, Sheaves B, Freeman D (2019). Sleep Disorders in Early Psychosis: Incidence, Severity, and Association With Clinical Symptoms. Schizophr Bull.

[R180] Reeve S, Sheaves B, Freeman D (2015). The role of sleep dysfunction in the occurrence of delusions and hallucinations: A systematic review. Clin Psychol Rev.

[R181] Rehman A, Waite F, Sheaves B, Biello S, Freeman D, Gumley A (2017). Clinician perceptions of sleep problems, and their treatment, in patients with non-affective psychosis. Psychosis.

[R182] Robinson JL, Erath SA, Kana RK, El-Sheikh M (2018). Neurophysiological differences in the adolescent brain following a single night of restricted sleep – A 7T fMRI study. Developmental Cognitive Neuroscience.

[R183] Rosenberg DR, Lewis DA (1995). Postnatal maturation of the dopaminergic innervation of monkey prefrontal and motor cortices: A tyrosine hydroxylase immunohistochemical analysis. Journal of Comparative Neurology.

[R184] Russell K, Rasmussen S, Hunter SC (2018). Insomnia and Nightmares as Markers of Risk for Suicidal Ideation in Young People: Investigating the Role of Defeat and Entrapment. J Clin Sleep Med.

[R185] Sacchet MD, Gotlib IH (2017). Myelination of the brain in Major Depressive Disorder: An in vivo quantitative magnetic resonance imaging study. Sci Rep.

[R186] Sämann PG, Tully C, Spoormaker VI, Wetter TC, Holsboer F, Wehrle R, Czisch M (2010). Increased sleep pressure reduces resting state functional connectivity. Magn Reson Mater Phy.

[R187] Sánchez MM, Hearn EF, Do D, Rilling JK, Herndon JG (1998). Differential rearing affects corpus callosum size and cognitive function of rhesus monkeys. Brain Research.

[R188] Sawyer SM, Azzopardi PS, Wickremarathne D, Patton GC (2018). The age of adolescence. The Lancet Child & Adolescent Health.

[R189] Schalbetter SM, von Arx AS, Cruz-Ochoa N, Dawson K, Ivanov A, Mueller FS, Lin H-Y, Amport R, Mildenberger W, Mattei D, Beule D (2022). Adolescence is a sensitive period for prefrontal microglia to act on cognitive development. Sci Adv.

[R190] Schroder CM, Malow BA, Maras A, Melmed RD, Findling RL, Breddy J, Nir T, Shahmoon S, Zisapel N, Gringras P (2019). Pediatric Prolonged-Release Melatonin for Sleep in Children with Autism Spectrum Disorder: Impact on Child Behavior and Caregiver’s Quality of Life. J Autism Dev Disord.

[R191] Shansky RM, Murphy AZ (2021). Considering sex as a biological variable will require a global shift in science culture. Nat Neurosci.

[R192] Sherman LE, Rudie JD, Pfeifer JH, Masten CL, McNealy K, Dapretto M (2014). Development of the Default Mode and Central Executive Networks across early adolescence: A longitudinal study. Developmental Cognitive Neuroscience.

[R193] Short MA, Weber N (2018). Sleep duration and risk-taking in adolescents: A systematic review and meta-analysis. Sleep Med Rev.

[R194] Sipe GO, Lowery RL, Tremblay MÉ, Kelly EA, Lamantia CE, Majewska AK (2016). Microglial P2Y12 is necessary for synaptic plasticity in mouse visual cortex. Nat Commun.

[R195] Skene NG, Roy M, Grant SG (2017). A genomic lifespan program that reorganises the young adult brain is targeted in schizophrenia. eLife.

[R196] Solmi M, Radua J, Olivola M, Croce E, Soardo L, Salazar de Pablo G, Il Shin J, Kirkbride JB, Jones P, Kim JH, Kim JY (2021). Age at onset of mental disorders worldwide: large-scale meta-analysis of 192 epidemiological studies. Mol Psychiatry.

[R197] Sousa SS, Amaro E, Crego A, Gonçalves ÓF, Sampaio A (2018). Developmental trajectory of the prefrontal cortex: a systematic review of diffusion tensor imaging studies. Brain Imaging and Behavior.

[R198] Sowell ER, Thompson PM, Holmes CJ, Jernigan TL, Toga AW (1999). In vivo evidence for post-adolescent brain maturation in frontal and striatal regions. Nat Neurosci.

[R199] Spear LP (2000). The adolescent brain and age-related behavioral manifestations. Neuroscience & Biobehavioral Reviews.

[R200] Stevens B (2008). Neuron-Astrocyte Signaling in the Development and Plasticity of Neural Circuits. NSG.

[R201] Suh S, Kim H, Dang-Vu TT, Joo E, Shin C (2016). Cortical Thinning and Altered Cortico-Cortical Structural Covariance of the Default Mode Network in Patients with Persistent Insomnia Symptoms. Sleep.

[R202] Swartz JR, Carrasco M, Wiggins JL, Thomason ME, Monk CS (2014). Age-related changes in the structure and function of prefrontal cortex-amygdala circuitry in children and adolescents: A multi-modal imaging approach. Neuroimage.

[R203] Sydnor VJ, Larsen B, Bassett DS, Alexander-Bloch A, Fair DA, Liston C, Mackey AP, Milham MP, Pines A, Roalf DR, Seidlitz J (2021). Neurodevelopment of the association cortices: Patterns, mechanisms, and implications for psychopathology. Neuron.

[R204] Takesian AE, Hensch TK (2013). Balancing plasticity/stability across brain development. Prog Brain Res.

[R205] Talbot LS, McGlinchey EL, Kaplan KA, Dahl RE, Harvey AG (2010). Sleep deprivation in adolescents and adults: changes in affect. Emotion.

[R206] Tarokh L, Carskadon MA, Achermann P (2010). Developmental Changes in Brain Connectivity Assessed Using the Sleep EEG. Neuroscience.

[R207] Tarokh L, Saletin JM, Carskadon MA (2016). Sleep in adolescence: Physiology, cognition and mental health. Neurosci Biobehav Rev.

[R208] Tashjian SM, Goldenberg D, Monti MM, Galván A (2018). Sleep quality and adolescent default mode network connectivity. Soc Cogn Affect Neurosci.

[R209] Telzer EH, Goldenberg D, Fuligni AJ, Lieberman MD, Gálvan A (2015). Sleep variability in adolescence is associated with altered brain development. Dev Cogn Neurosci.

[R210] Thompson A, Lereya ST, Lewis G, Zammit S, Fisher HL, Wolke D (2015). Childhood sleep disturbance and risk of psychotic experiences at 18: UK birth cohort. Br J Psychiatry.

[R211] Thorisdottir IE, Asgeirsdottir BB, Kristjansson AL, Valdimarsdottir HB, Tolgyes EMJ, Sigfusson J, Allegrante JP, Sigfusdottir ID, Halldorsdottir T (2021). Depressive symptoms, mental wellbeing, and substance use among adolescents before and during the COVID-19 pandemic in Iceland: a longitudinal, population-based study. The Lancet Psychiatry.

[R212] Tomasella E, Bechelli L, Ogando MB, Mininni C, Guilmi MND, Fino FD, Zanutto S, Elgoyhen AB, Marin-Burgin A, Gelman DM (2018). Deletion of dopamine D2 receptors from parvalbumin interneurons in mouse causes schizophrenia-like phenotypes. PNAS.

[R213] Tseng K-Y, O’Donnell P (2007). Dopamine modulation of prefrontal cortical interneurons changes during adolescence. Cereb Cortex.

[R214] Tseng KY, O’Donnell P (2005). Post-pubertal Emergence of Prefrontal Cortical Up States Induced by D1–NMDA Co-activation. Cereb Cortex.

[R215] Twenge JM, Cooper AB, Joiner TE, Duffy ME, Binau SG (2019). Age, period, and cohort trends in mood disorder indicators and suicide-related outcomes in a nationally representative dataset, 2005–2017. Journal of Abnormal Psychology.

[R216] Uhlhaas PJ, Singer W (2010). Abnormal neural oscillations and synchrony in schizophrenia. Nat Rev Neurosci.

[R217] Uliana DL, Gomes FV, Grace AA (2020). Prelimbic medial prefrontal cortex disruption during adolescence increases susceptibility to helpless behavior in adult rats. European Neuropsychopharmacology.

[R218] UNICEF (2021). The State of the World’s Children 2021.

[R219] Urrila AS, Artiges E, Massicotte J, Miranda R, Vulser H, Bézivin-Frere P, Lapidaire W, Lemaître H, Penttilä J, Conrod PJ, Garavan H (2017). Sleep habits, academic performance, and the adolescent brain structure. Sci Rep.

[R220] Van DTR, Becker SP, Byars KC (2019). Rates of Mental Health Symptoms and Associations With Self-Reported Sleep Quality and Sleep Hygiene in Adolescents Presenting for Insomnia Treatment. Journal of Clinical Sleep Medicine.

[R221] Vijayakumar N, Bartholomeusz C, Whitford T, Hermens DF, Nelson B, Rice S, Whittle S, Pantelis C, McGorry P, Schäfer MR, Amminger GP (2016). White matter integrity in individuals at ultra-high risk for psychosis: a systematic review and discussion of the role of polyunsaturated fatty acids. BMC Psychiatry.

[R222] Waite F, Bradley J, Chadwick E, Reeve S, Bird JC, Freeman D (2018). The Experience of Sleep Problems and Their Treatment in Young People at Ultra-High Risk of Psychosis: A Thematic Analysis. Front Psychiatry.

[R223] Walker DM, Bell MR, Flores C, Gulley JM, Willing J, Paul MJ (2017). Adolescence and Reward: Making Sense of Neural and Behavioral Changes Amid the Chaos. J Neurosci.

[R224] Wang H-X, Gao W-J (2009). Cell-type Specific Development of NMDA Receptors in the Interneurons of Rat Prefrontal Cortex. Neuropsychopharmacology.

[R225] Wassing R, Schalkwijk F, Lakbila-Kamal O, Ramautar JR, Stoffers D, Mutsaerts HJMM, Talamini LM, Van Someren EJW (2019). Haunted by the past: old emotions remain salient in insomnia disorder. Brain.

[R226] Weaver MD, Barger LK, Malone SK, Anderson LS, Klerman EB (2018). Dose-Dependent Associations Between Sleep Duration and Unsafe Behaviors Among US High School Students. JAMA Pediatr.

[R227] Willing J, Juraska JM (2015). The Timing of Neuronal Loss Across Adolescence in the Medial Prefrontal Cortex of Male and Female Rats. Neuroscience.

[R228] Wohleb ES, Terwilliger R, Duman CH, Duman RS (2018). Stress-induced neuronal CSF1 provokes microglia-mediated neuronal remodeling and depressive-like behavior. Biol Psychiatry.

[R229] World Health Organisation (2011). Global burden of mental disorders and the need for a comprehensive, coordinated response from health and social sectors at the country level.

[R230] World Health Organisation (1965). Health problems of adolescents. Technical report series.

[R231] Yaeger CE, Ringach DL, Trachtenberg JT (2019). Neuromodulatory control of localized dendritic spiking in critical period cortex. Nature.

[R232] Zhang L, Cui Z, Sasser J, Oshri A (2021). 222 COVID-19 Related Stress Intensify the Impact of Child Maltreatment on Sleep Quality. Sleep.

[R233] Zhang Y-Q, Lin W-P, Huang L-P, Zhao B, Zhang C-C, Yin D-M (2021). Dopamine D2 receptor regulates cortical synaptic pruning in rodents. Nat Commun.

[R234] Zhou Y, Lai CSW, Bai Y, Li W, Zhao R, Yang G, Frank MG, Gan W-B (2020). REM sleep promotes experience-dependent dendritic spine elimination in the mouse cortex. Nat Commun.

