# Supplementary material for: Adolescent sleep and the foundations of prefrontal cortical development and dysfunction
Source: Prog Neurobiol. Author manuscript; Available in PMC 2024 Jul 12. (PMC7616212; doi:10.1016/j.pneurobio.2022.102338)
Supplement: Appendix [file EMS197318-supplement-Appendix.pdf]

APPENDIX A: Defining adolescence

What is adolescence?

Adolescence can be defined as the period spanning the transition from childhood to adulthood, characterized by extensive physical, neurodevelopmental, psychological, and social changes (Spear, 2000). However, most of these changes occur gradually, and temporally overlap to some extent. The onset of adolescence is often linked to puberty and associated changes in activation of the neuroendocrine hypothalamic–pituitary–gonadal axis, the timing of which is variable between sexes; its end is linked to cognitive maturation and achieving social and cultural norms such as marriage and parenting. Although adolescence typically encompasses the period from 10 to 20 years of age (World Health Organisation, 1965) individual start and end points can therefore vary. Adolescence can be further divided into early (from 10 to 13 years), middle (from 14 to 16 years) and late adolescence (from 17 to 20 years). Early adolescence is characterized by pubertal development, involving rapid increases in hormone levels, accelerated growth, and development of secondary sexual characteristics. Post-pubertal maturation constitutes "proper" adolescence, which is accompanied by cognitive maturation, personality development and acquisition of appropriate social behaviours. Defining the endpoint of adolescence is difficult considering how physical and cognitive maturation continue into the thirties and acquisition of economic independence and social milestones that mark the transition to adulthood, such as parenting and marriage, are influenced by society. Recently it has been suggested that the period of adolescence be extended to 24 years of age, better reflecting modern society where social, emotional, and economical independence are often achieved later in life than in previous generations (Sawyer et al., 2018).

Adolescence is observed across many species used in biomedical research (Spear, 2000). In the macaque monkey adolescence encompasses the period between 2 to 4 years of age (Lewis, 1997). In rodents, adolescence covers the postnatal period from age 21 to 60 days (P21-P60), and can be further divided in early adolescence, postnatal day (P) 21 to 34 (P21-P34), middle adolescence, around the onset of puberty (P35-P45), and late adolescence from P46 to P60 (Laviola et al., 2003). Although certain aspects of adolescence are unique to humans, rodents also show characteristic physical, neurodevelopmental and behavioural (including chronobiological) changes homologous to those observed in humans, supporting face and construct validity of animal models to study psychobiological processes during adolescence (Spear, 2000). Adolescent rodents display changes in growth rates, maturation of the reproductive system, as well as brain maturation and behavioral changes seen in humans, such as the emergence of sexually oriented partner-seeking, elevated levels of social behaviours, sensation-seeking and risk-taking exploration. Rodents therefore represent excellent models to study the shifting neurobiology of adolescence at the cellular and systems level.

APPENDIX B: Sleep disruption and stress

Acute versus Chronic sleep loss

Sleep's effects on the brain depend on the amount of sleep lost. The term "acute" is typically used when describing one or two nights of sleep restriction or deprivation, while "chronic" is used to describe longer periods of restricted or disrupted sleep, typically lasting 4-7 days in controlled laboratory settings (Lemola et al., 2013; Philip et al., 2012), or potentially over the course of many years in longitudinal studies tracking patients in their home environment (Breslau et al., 1996). A major challenge when comparing across studies is that the duration and severity of sleep interventions vary considerably. Moreover, the magnitude of the observed effect will vary based on when the tests are performed, with more severe deficits

observed if testing is performed during the subjects night-time period (Basner et al., 2013). Although studies are regularly reported as either chronic or acute, research shows that sleep restriction across days results in near-linear declines in behavioural alertness and cognitive performance (Basner et al., 2013; Belenky et al., 2003). Similarly, recovery from sleep restriction occurs in a fairly linear, dose-dependent fashion (Banks et al., 2010). However, it is likely that the relative rate of decline and subsequent recovery will vary based on the cognitive, behavioural or neurophysiological variable under investigation. A significant corollary of the data presented in this review is that while acute effects are easier to establish and study in the laboratory setting, it is chronic effects that will best inform out understanding of adolescent mental health in the context of societal pressures towards reduced sleep. This is further supported by observations that the cumulative waking deficits of chronic sleep restriction can resemble the impairments in vigilant attention performance observed in the most severe cases of total sleep deprivation, and are likely related to long-term changes in neuromodulatory function and brain connectivity (Basner et al., 2013).

Sleep and stress

Sleep loss and stress are inherently linked, with any attempt to enforce sustained wake leading to "stress" comprising a complex physiological response to re-establish homeostasis. Sleep deprivation perturbs the hypothalamic pituitary axis, resulting in, among other physiological modifications, increased corticosterone. Therefore, distinguishing the effects of stress from those due to lack of sleep can be challenging. Experimentally, it is nearly impossible to study the "pure" effects of sleep loss and perform a stress-free, total sleep deprivation. However, animal studies reveal how many molecular and behavioural changes observed following sleep deprivation persist in animals subjected to adrenalectomy, suggesting that these changes are independent of the adrenal stress response (Mongrain et al., 2010). In addition, similar molecular changes in animals normally occur during spontaneous wake (Cirelli et al., 2004), indicating baseline stress associated with spontaneous waking relative to sleep, although not as high as after sleep deprivation. Furthermore, it is known that psychological stressors impair sleep. In controlled experimental settings, mild stressors lead to heightened physiological arousal with decreases in slow wave sleep, REM sleep, and sleep efficiency (Kim and Dimsdale, 2007). Therefore, psychological stressors can deteriorate sleep, thus further promoting the release of stress hormones and establish a vicious circle with potentially deleterious effects on mental health.

APPENDIX C: Sleep during the COVID-19 pandemic

The Impacts of COVID-19

Since 2019, COVID-19 has taken hold of our daily lives and challenged our mental health. Amongst teenagers, consequent disruption has had pronounced, but often diverse, effects on sleep. Across many studies average sleep metrics actually improved in the adolescent population, with the difference between weekday and weekend sleep duration, so-called "social jetlag", markedly reduced (Gruber et al., 2021, 2020; Lian et al., 2021). This change was seemingly driven by later school start times, the absence of daily school commutes due to online teaching and reduced after-school activities impacting time available for homework (Gruber et al., 2021). These studies indicate how adjustments to school hours can benefit adolescents by reducing daytime sleepiness, increasing overall sleep and potentially benefit adolescent brain development and function. However, the recent pandemic was a stressful and traumatic time and, as with many other societal factors, may have disproportionately affected those already struggling to cope. For example, sleep quality was significantly reduced in a subpopulation of adolescents who displayed higher levels of perceived stress (Gruber et al., 2021). For some, being kept at home also meant increased exposure to threats and abuse. Those exposed to childhood maltreatment displayed increased levels of sleep disturbances,

which were exacerbated by COVID-related stress (L. Zhang et al., 2021). Similar COVID-related deficits in sleep health were also observed in patients with ADHD (Bruni et al., 2021). There is already evidence that COVID-19 has negatively impacted adolescent mental health (Thorisdottir et al., 2021), perhaps accentuating a pre-existing global decline in adolescent mental well-being. In the long-term it is likely that the detrimental consequences of COVID-19 on adolescents will be considerable, particularly amongst those already at elevated risk of developing mental health disorders. Minimising the human cost of COVID-19 is not only a moral necessity but also of significant benefit to the economy and wider society, with UNICEF estimating that failure to invest in adolescent mental health costs US$387 billion in lost human potential and contributions to the future economy (UNICEF, 2021). Based on the findings presented in this review, taking steps to promote adolescent sleep health may offer a cost-effective and globally scalable method of reducing some of the burden on adolescent mental health, helping improve lifelong outcomes.
